# Type 1 and Type 2 Epstein-Barr viruses induce proliferation, and inhibit differentiation, in infected telomerase-immortalized normal oral keratinocytes

**DOI:** 10.1371/journal.ppat.1010868

**Published:** 2022-10-03

**Authors:** Deo R. Singh, Scott E. Nelson, Abigail S. Pawelski, Juan A. Cantres-Velez, Alisha S. Kansra, Nicholas P. Pauly, Jillian A. Bristol, Mitchell Hayes, Makoto Ohashi, Alejandro Casco, Denis Lee, Stuart A. Fogarty, Paul F. Lambert, Eric C. Johannsen, Shannon C. Kenney

**Affiliations:** 1 Department of Oncology, School of Medicine and Public Health, University of Wisconsin- Madison, Madison, Wisconsin, United States of America; 2 Department of Medicine, School of Medicine and Public Health, University of Wisconsin-Madison, Madison, Wisconsin, United States of America; Pennsylvania State University College of Medicine, UNITED STATES

## Abstract

Differentiated epithelial cells are an important source of infectious EBV virions in human saliva, and latent Epstein-Barr virus (EBV) infection is strongly associated with the epithelial cell tumor, nasopharyngeal carcinoma (NPC). However, it has been difficult to model how EBV contributes to NPC, since EBV has not been shown to enhance proliferation of epithelial cells in monolayer culture *in vitro* and is not stably maintained in epithelial cells without antibiotic selection. In addition, although there are two major types of EBV (type 1 (T1) and type 2 (T2)), it is currently unknown whether T1 and T2 EBV behave differently in epithelial cells. Here we inserted a G418 resistance gene into the T2 EBV strain, AG876, allowing us to compare the phenotypes of T1 Akata virus versus T2 AG876 virus in a telomerase-immortalized normal oral keratinocyte cell line (NOKs) using a variety of different methods, including RNA-seq analysis, proliferation assays, immunoblot analyses, and air-liquid interface culture. We show that both T1 Akata virus infection and T2 AG876 virus infection of NOKs induce cellular proliferation, and inhibit spontaneous differentiation, in comparison to the uninfected cells when cells are grown without supplemental growth factors in monolayer culture. T1 EBV and T2 EBV also have a similar ability to induce epithelial-to-mesenchymal (EMT) transition and activate canonical and non-canonical NF-κB signaling in infected NOKs. In contrast to our recent results in EBV-infected lymphoblastoid cells (in which T2 EBV infection is much more lytic than T1 EBV infection), we find that NOKs infected with T1 and T2 EBV respond similarly to lytic inducing agents such as TPA treatment or differentiation. These results suggest that T1 and T2 EBV have similar phenotypes in infected epithelial cells, with both EBV types enhancing cellular proliferation and inhibiting differentiation when growth factors are limiting.

## Author summary

Although EBV infection is required for the development of undifferentiated NPC in humans, it previously has not been possible to show that EBV infection enhances epithelial cell proliferation *in vitro* in undifferentiated cells. In addition, while there are two major types of EBV (T1 andT2), and T1 and T2 EBV behave differently in B cells, it is not known whether T1 and T2 EBV infection behave differently in epithelial cells. Here we have inserted a G418R/GFP cassette into the non-essential BXLF1 gene of AG876 T2 EBV strain to select for normal oral keratinocyte cells (NOKs) stably infected with T2 AG876 virus, and to compare their phenotype to NOKs cells infected with T1 Akata virus. We find that both viruses induce cellular proliferation, and suppress differentiation, in NOKs when growth factors are severely limiting, and have similar levels of lytic reactivation in response to TPA and differentiation. The ability of EBV infection to activate cellular proliferation and inhibit differentiation under growth factor-restricted conditions likely contributes to NPC development.

## Introduction

Epstein-Barr virus (EBV) is a cancer-associated gamma herpesvirus that infects the majority of adult humans and causes both B-cell and epithelial-cell malignancies, including Burkitt lymphoma, diffuse large B cell lymphoma, Hodgkin lymphoma, gastric carcinoma, and undifferentiated nasopharyngeal carcinoma (NPC) [1–3]. EBV, like all herpes viruses, persists in the host for life and infects cells in either latent or lytic forms. The major reservoir for latent EBV infection in humans is memory B cells, while differentiated oropharyngeal epithelial cells are a major site of lytic viral infection [3–5]. Although EBV can express up to 9 different latent viral proteins in latently infected B cells, during latency no infectious viral particles are produced and the viral genome is replicated by the host cell-encoded DNA polymerase [4]. In lytically infected cells, the virus is replicated using the virally-encoded DNA polymerase and infectious virions are produced, allowing spread of the virus from cell-to-cell and host-to-host [3,6,7]. EBV infection of normal oropharyngeal epithelial cells in humans is primarily lytic [8,9] and is the major source of infectious EBV virions in saliva [5]. A clinical syndrome known as oral hairy leukoplakia (OHL), that primarily occurs in immunocompromised patients, is due to completely lytic EBV infection of non-transformed, differentiated tongue epithelial cells without co-existing latent infection [8,10]. However, while latent and lytic EBV infection both contribute to the early development of EBV-induced cancers, by the time tumors are fully developed they are largely composed of latently infected cells [6]. Thus, the EBV-associated epithelial cell tumor, undifferentiated nasopharyngeal carcinoma, primarily has the latent form of EBV infection [11].

How EBV achieves latency in normal epithelial cells is still not completely understood but increasing evidence from our labs and others suggests that cellular transcription factors (including KLF4 and BLIMP1) which are turned on by, and required for, epithelial cell differentiation are used by the virus to activate its two immediate-early (IE) genes (BZLF1 and BRLF1) and ensure that the virus lytically reactivates in differentiated epithelial cells [12,13]. Conversely, we recently showed that the master regulator of undifferentiated basal epithelial cell identity, ΔNp63ɑ, inhibits lytic EBV reactivation in undifferentiated basal epithelial cells by blocking activity of the BZLF1 IE promoter [14]. Once the two EBV IE proteins are expressed, they transcriptionally activate other lytic viral genes required for the lytic form of viral DNA replication and virion production [3,7].

There are two different types of EBV, Type 1 (T1) and Type 2 (T2) [15]. T2 EBV transforms B cells into lymphoblastoid cell lines *in vitro* less efficiently than T1 EBV, and may be under-represented in endemic Burkitt lymphomas [16], although it is equally transforming as type 1 EBV in humanized mouse models and appears to be similar in its ability to cause other types of human lymphomas [17–28]. Of note, we recently discovered that T2 EBV is more lytic than T1 EBV in newly transformed B cell lines and in humanized mice [27,29]. However almost nothing is known about differences between T1 and T2 EBV infection in epithelial cells. T1 EBV infection is much more common than T2 EBV infection in western countries [30] and most previous studies in the EBV field have been performed using T1 EBV strains. T2 EBV infection has been reported to be present in up to 50% of the population in sub-Saharan Africa and New Guinea [15–22], and humans can be simultaneously infected with both types [21,22]. The most divergent genes in the T1 versus T2 EBV genomes are the EBNA2 and EBNA 3A/B/C latency genes [23]. Although the EBNA2 and EBNA3A/B/C proteins are expressed in EBV infected B cells with “type III” viral latency, and the EBNA2 and EBNA3C viral proteins are required for EBV transformation of B cells *in vitro* [4], the EBNA2 and EBNA3A/B/C proteins are not thought to be usually expressed in latently or lytically infected epithelial cells, although one paper reported EBNA2 expression in oral hairy leukoplakia lesions [31].

Another difference between T1 EBV and T2 EBV is that all T2 EBV strains contain the Zp-V3 form of the promoter (Zp) driving the BZLF1 immediate-early gene, while most T1 strains have the “prototype” Zp-P form [17,18]. We have shown that efficient B-cell receptor (BCR)-mediated lytic EBV reactivation in EBV+ B cells requires an NFATc1 binding site that is present on the Zp-V3 form of the BZLF1 promoter (but not the prototype Zp-P form) [32]. Furthermore, we recently showed that the higher level of constitutive lytic infection in early passage LCLs with T2 versus T1 EBV infection is due not only to the universal presence of the NFATc1-responsive form of the Zp promoter (Zp-V3) in all T2 strains, but also a much higher level of the activated forms of both NFATc1 and NFATc2 in T2 LCLs [27]. However, some T1 EBV strains, including the Akata strain, contain the Zp-V3 form of the BZLF1 promoter, and it is not known whether NFAT family members are involved in regulating lytic EBV reactivation in epithelial cells.

NOKs is a telomerase-immortalized normal oral keratinocyte cell line that can be selected for sustained latent EBV infection using G418 selection, and which retains the ability to differentiate [33–36]. We previously showed that infection of NOKs with the type 1 Akata EBV strain inhibits differentiation of NOKs grown (“rafted”) on air-liquid interface culture or differentiated by suspending the cells in methylcellulose [34]. In addition, we demonstrated that lytic reactivation of EBV occurs only in the differentiated layers of rafted NOKs-Akata cells [12,13,14]. However, in past studies, we and others did not observe substantial differences in the proliferative phenotypes of EBV-infected versus uninfected NOKs grown in monolayer culture [33,35]. Here we have defined growth-restricted culture conditions in which the ability of EBV infection to induce cellular proliferation and inhibit spontaneous differentiation of NOKs in monolayer cultures can be consistently and clearly observed. In contrast, our previously published studies may have missed the growth promoting, and differentiation inhibiting, effects of EBV in monolayer NOKs cultures due to the presence of high levels of epidermal growth factor (EGF) and bovine pituitary extract (BPE) in the media.

In this study, we have used homologous recombination to insert a G418R/GFP expression cassette into the nonessential BXLF1 gene in the type 2 AG876 virus genome. Using this new G418R AG876 virus, we compared the phenotypes of uninfected NOKs cells, NOKs cells stably infected with type 2 AG876 virus and NOKs stably infected with type 1 Akata virus (containing the same G418R/GFP gene cassette inserted into its BXLF1 gene) [36]. To our knowledge, this is the first study to compare T1 versus T2 EBV infection in epithelial cells. We show that both types of EBV promote proliferation, inhibit epithelial cell differentiation and induce an EMT phenotype in NOKs. We also find similar levels of lytic EBV reactivation in NOKs infected with each EBV virus type. Thus, the phenotypes of T1 versus T2 EBV are more similar in epithelial cells than in B cells.

## Results

### Creation of NOKs lines stably infected with a G418-resistant Type 2 AG876 EBV strain or G418-resistant Type 1 Akata EBV strain

To create a type 2 EBV virus that can stably infect epithelial cells (and be titered), we inserted a GFP/G418R gene cassette (PCR amplified from the Akata virus genome in the BX1 BL line [37]) by homologous recombination into the nonessential BXLF1 (TK) gene in the type 2 EBV genome within AG876 Burkitt lymphoma (BL) cells. After selection, G418-resistant AG876 BL cells were induced to lytically reactivate by treating cells with TPA and sodium butyrate, co-cultured with HeLa cells for several days, and then EBV-infected HeLa cell clones were obtained using G418 selection. The G418R AG876 virus was then lytically reactivated from infected HeLa cell clones by transfecting cells with BZLF1 and BRLF1 (encoding the two EBV IE proteins, Z and R) expression vectors, and infectious virion particles from the supernatant were used to stably infect EBV-negative Akata Burkitt lymphoma (BL) cells using G418 selection. BL cells stably infected with GFP+/G418R AG876 virus were then lytically reactivated by treatment with TPA/sodium butyrate, and co-cultured with uninfected NOKs for several days before removing the BL cells and then selecting for type 2 AG876 EBV-infected NOKs using G418 selection. Type 1 Akata EBV-infected NOKs were obtained by co-culturing NOKs for several days with lytically induced BX1 BL cells (infected with a GFP+/G418R Akata virus type 1 strain [37]), removing the BL cells, and then selecting for G418 resistant NOKs lines. Two different uninfected NOKs lines (“NOKs-1” and “NOKs-2”) that had been separately maintained in two different laboratories were each infected with both virus types; similar results were obtained in each of the two NOKs lines.

### NOKs infected with type 1 Akata EBV and type 2 AG876 EBV have similar cellular gene expression patterns

To compare the effects of AG876 virus versus Akata virus infection on cellular and viral gene expression in NOKs, we harvested RNA from three separate samples each of uninfected NOKs, or NOKs stably infected with either type 2 AG876 or type 1 Akata viruses and performed RNA-seq analysis. Of note, cells were grown using only low levels of EGF (0.2 ng/mL) and 12.5mg of bovine pituitary extract and then starved for 24 hours with no EGF or bovine pituitary extract prior to harvesting RNA in this experiment.

As shown in Table 1, comparison of cellular gene expression in the T1 Akata virus-infected NOKs versus T2 AG876 virus-infected NOKs reveals 85 genes were significantly upregulated, and 36 genes significantly downregulated, in the Akata versus AG876 virus infected cells. In contrast, we previously found that close to 600 cellular genes were expressed at significantly different levels in T1 versus T2 EBV-infected lymphoblastoid B cell lines [29]. The AG876 virus-infected and Akata virus-infected NOKs are much more similar to each other compared to the uninfected NOKs. AG876 virus-infected NOKs have 269 upregulated genes and 755 downregulated genes relative to the uninfected NOKs, while the Akata virus-infected NOKs have 308 upregulated genes and 726 downregulated genes relative to the uninfected cells (Table 1). S1–S3 Figs show heat maps of the top 100 differentially regulated genes in T1 Akata virus-infected cells versus T2 AG876 virus-infected NOKs, in AG876 virus-infected versus uninfected NOKs, and in Akata virus-infected versus uninfected NOKs, respectively. Bulk RNA-seq data of Type 1 EBV-infected, Type 2 EBV-infected and uninfected NOKs are shown in S1 Table.

**Table 1:** Differentially expressed genes measured in bulk RNA-seq. The number of cellular genes showing at least a two-fold change in gene expression is shown when comparing either NOKs-Akata (Type1) to NOKs-AG876 (Type 2), NOKs-Akata to uninfected NOKs, or NOKs-AG876 to uninfected NOKs.

Comparison	Upregulated	Downregulated
Akata vs. AG876	85	35
Akata vs. Uninfected	308	726
AG876 vs. Uninfected	259	755

Fold change >2, FDR <0.05

### T1 Akata virus-infected and T2 AG876 virus-infected NOKs both have RNA-seq expression signatures suggesting increased proliferation and decreased differentiation compared to uninfected NOKs

**Fig 1:**
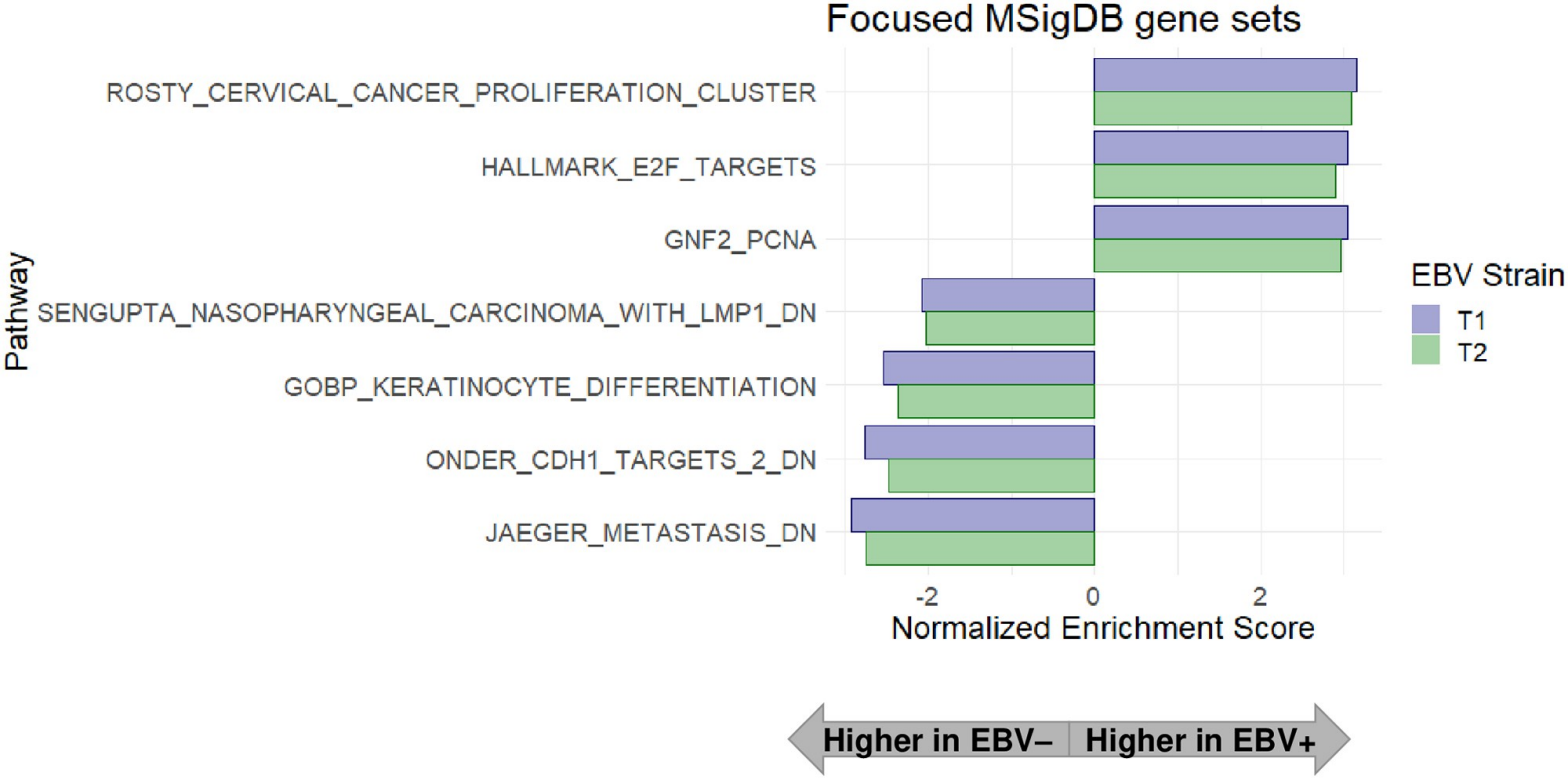
Gene set enrichment analysis (GSEA) suggests increased proliferation, decreased keratinocyte differentiation and decreased E-cadherin signaling in both the type 1 EBV-infected and type 2 EBV-infected NOKs in comparison to uninfected NOKs when growth factors are limiting. AG876 virus-infected, Akata virus-infected, or uninfected NOKs were grown in growth factor-restricted conditions, and RNA-seq and GSEA were performed as described in the Materials and Methods sections. Displayed are GSEA results on a focused set of differentially expressed pathways related to EBV-infected versus uninfected NOKs following interrogation of extensive GSEA results shown in S4 Fig. Displayed pathways contain a Benjamini-Hocheberg (BH)-adjusted p-value of <0.05 and are sorted by Normalized Enrichment Scores (NES). Pathways upregulated in the EBV-infected NOKs relative to uninfected NOKs are associated with NES values greater than 0, and down-regulated pathways are associated with NES values less than 0.

To identify signaling pathways regulated by T2 AG876 virus infection and/or T1 Akata virus infection in NOKs, we analyzed the RNA-seq data using Gene Set Enrichment Analysis (GSEA). As shown in Fig 1 and S4 Fig, in comparison to the uninfected NOKs, both T2 AG876 virus-infected and T1 Akata virus-infected NOKs have increased expression of genes in the “HALLMARK_E2F_TARGETS” gene set, suggesting enhanced proliferation of both type 2 AG876 virus-infected and type 1 Akata virus-infected NOKs in comparison to the uninfected NOKs. Other gene sets suggestive of increased cellular proliferation, including the “ROSTY_CERVICAL_CANCER_PROLIFERATION_CLUSTER”, and “GNF2_PCNA” gene sets were likewise more highly expressed in both the type 2 AG876 virus-infected and type 1 Akata virus- infected NOKs.

Type 2 AG876 virus-infected and type 1 Akata virus-infected NOKs also shared similar patterns of down-regulated genes sets in comparison to the uninfected NOKs. In particular, multiple different gene sets associated with epithelial cell differentiation, including the “GO_KERATINOCYTE_ DIFFERENTIATION” gene set, were down-regulated in both the T1 Akata virus-infected and T2 AG876 virus-infected NOKs in comparison to the uninfected NOKs (Fig 1 and S4 Fig). Other interesting genes sets with down-regulated expression in both the AG876 virus-infected and Akata virus-infected NOKs in comparison to the uninfected NOKs were the “ONDER_CDH1_TARGETS_3_DN” gene set and the “JAEGER_METASTASIS_DN” gene set. The down-regulation of the latter two gene sets suggests decreased e-cadherin (CDH1) expression and increased metastatic potential in the type 1 and type 2 EBV infected NOKs in comparison to the uninfected NOKs. In addition, the “SENGUPTA_NASOPHARYNGEAL_CARCINOMA_LMP1_DOWN” gene set was also expressed at a lower level in both Akata virus-infected and AG876 virus-infected NOKs in comparison to the uninfected cells (Fig 1 and S4 Fig). Since this gene set consists of cellular genes that are more down-regulated in NPC tumors with high-level LMP1 compared to NPC tumors with low level or no LMP1 expression [38], this result suggests that a number of the down-regulated cellular genes in EBV-infected versus uninfected NOKs cells may be due to LMP1 expression.

### T1 Akata and T2 AG876 virus infected NOKs have similar patterns of EBV gene transcription and latent protein expression

The results of the RNA-seq analysis were also used to align viral transcripts to the T1 and T2 EBV genomes, allowing us to compare the levels of different viral transcripts in T1 versus T2 EBV-infected NOKs. As shown in S5 Fig this analysis revealed similar ratios of latent versus lytic viral transcripts in NOKs cells infected with T1 Akata and T1 AG876 virus, although the total number of all EBV-related transcripts was higher in the AG876 virus infected cells. As expected, the EBV genes that are most divergent in T1 versus T2 EBV (EBNA2 and EBNA3A/B/C) are not transcribed in Akata virus-infected or AG876 virus-infected NOKs. In addition, we performed immunoblot analyses of Akata- and AG876- infected NOKs to compare the expression patterns of EBV latency proteins. As shown in S6 Fig, NOKs infected with Akata and AG876 viruses express similar levels of the latent EBV LMP1 and EBNA1 proteins (although both LMP1 and EBNA1 expression in NOKs is considerably lower than that expressed in an EBV-infected lymphoblastoid cell line), and do not express the EBV EBNA2 protein, confirming that they have “type II” viral latency. Somewhat surprisingly, we could not detect expression of the latent LMP2A protein by immunoblot analyses in EBV-infected NOKs, although we could detect LMP2A in the lymphoblastoid cell line.

### Type 1 Akata and type 2 AG876 EBV infection both promote cellular proliferation of NOKs when growth factors are limiting

**Fig 2:**
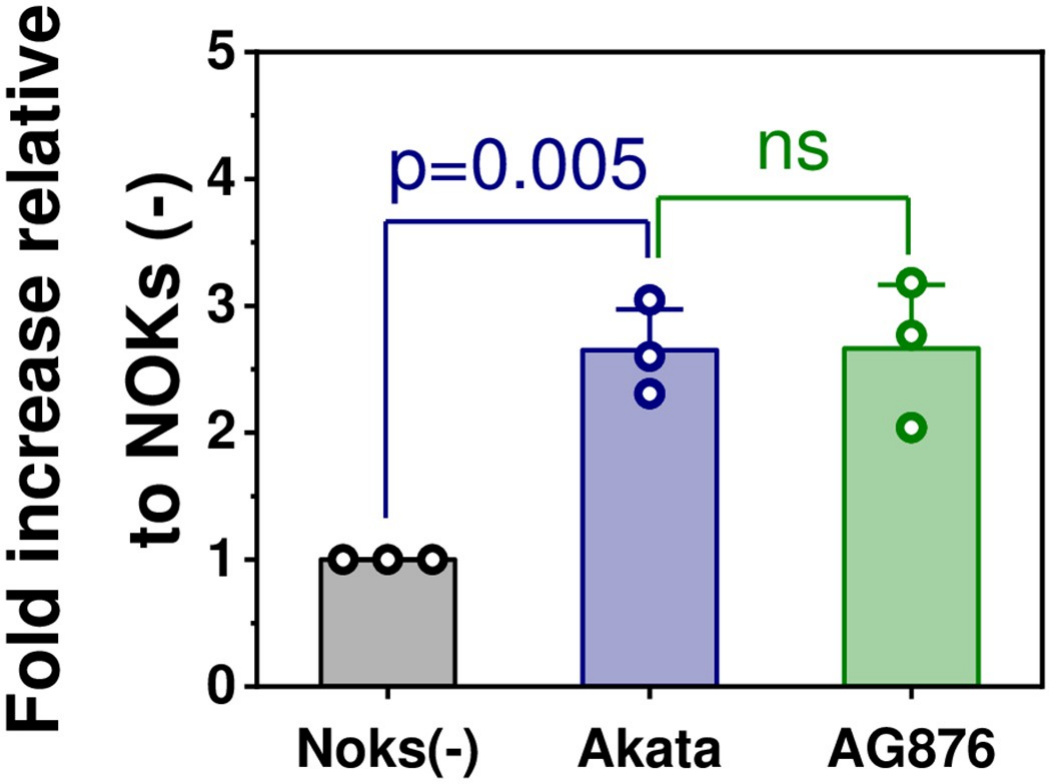
Both type 1 and type 2 EBV-infected NOKs proliferate faster than uninfected NOKs when growth factors are limiting. 50,000 uninfected NOKs, Type 1 Akata EBV-infected NOKs, or Type 2 AG876 EBV-infected NOKs (each in the context of the “NOKs-2” line) were uniformly seeded in each well of a 6 well plate, and then grown in KSFM medium without any EGF or BPE supplement. After 5 days, cells were counted using trypan blue staining. The total cells obtained from each Akata EBV-infected or AG876 EBV-infected NOKs condition was normalized to the number of cells obtained from uninfected NOKs conditions. The normalized data was plotted as a bar chart. Individual data points represent the fold- growth increased relative to uninfected NOKs (set as 1). The bars represent the average value of fold-increase relative to uninfected NOKs. The error bars represent the standard error. Statistical analysis was performed using one-way ANOVA. p<0.05 was considered statistically significant.

**Fig 3:**
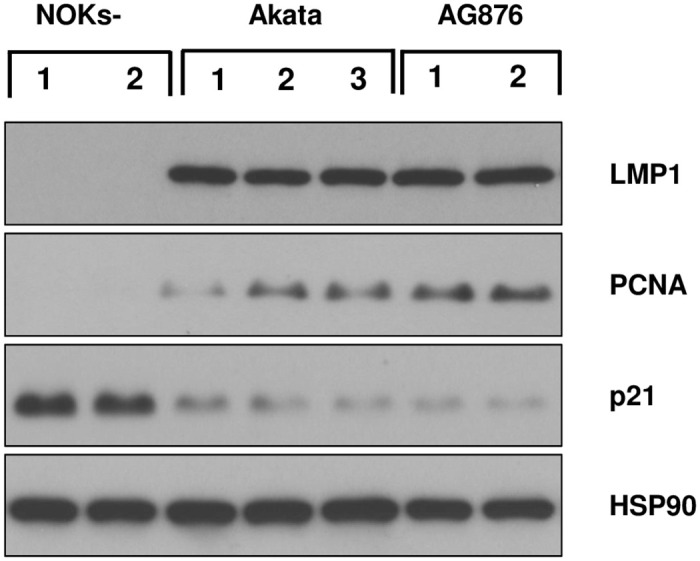
Type 1 Akata virus and type 2 AG876 virus both induce proliferation in NOKs when growth factors are limiting. Uninfected, Akata EBV-infected, or AG876 EBV-infected NOKs (each in the context of the “NOKs 2” line) were seeded in triplicate (125K cells per well) in 6 well plates and grown in KSFM medium without supplements for 24 hours. Immunoblot analysis was then performed to assess expression levels of LMP1, PCNA, p21, or HSP90 (loading control) as indicated.

We previously showed that Akata virus-infected NOKs contain abnormal suprabasal proliferating cells when “rafted” in air-liquid interface culture that promotes stratification of the epithelial cells and have an RNA-seq signature suggestive of increased proliferation and decreased differentiation when cells are differentiated by suspending the cells in methylcellulose. However, we did not previously observe an RNA-seq signature suggestive of enhanced proliferation and/or decreased differentiation when NOKs are grown in monolayer culture in the presence of EGF and bovine pituitary extract [34]. Likewise, the Scott laboratory reported that Akata virus-infected NOKs grown in monolayer culture with high levels of EGF and BPE proliferate similarly as the uninfected cells [35]. To determine if type 1 and/or type 2 EBV infection can increase NOKs proliferation when growth factors are severely limiting, equal numbers of uninfected, Akata virus-infected and AG876 virus-infected NOKs were plated at sub-confluent conditions in KSFM media the absence of EGF and bovine pituitary extract (or serum), and 5 days later the number of viable cells in each condition was determined by trypan blue staining. As shown in Fig 2, NOKs infected with either the Akata type 1 EBV strain, or the AG876 type 2 EBV strain, proliferated significantly more than uninfected NOKs in these growth factor-restricted conditions. In addition, when cells were plated at sub-confluent conditions and grown in the absence of growth factors, immunoblot analysis confirmed NOKs infected with either type 1 Akata virus or type 2 AG876 virus have increased expression of PCNA (a marker for cellular proliferation) and decreased p21 expression (a marker for cell cycle exit) compared to uninfected NOKs (Fig 3 and S7 Fig). These results reveal that EBV infection promotes proliferation of NOKs when growth factors are limiting, and that the effects of type 1 and type 2 EBV infection are similar in this regard.

### Type 2 AG876 virus infection and Type 1 Akata virus infection both induce an EMT phenotype in NOKs and activate the canonical and non-canonical NF-κB pathways

**Fig 4:**
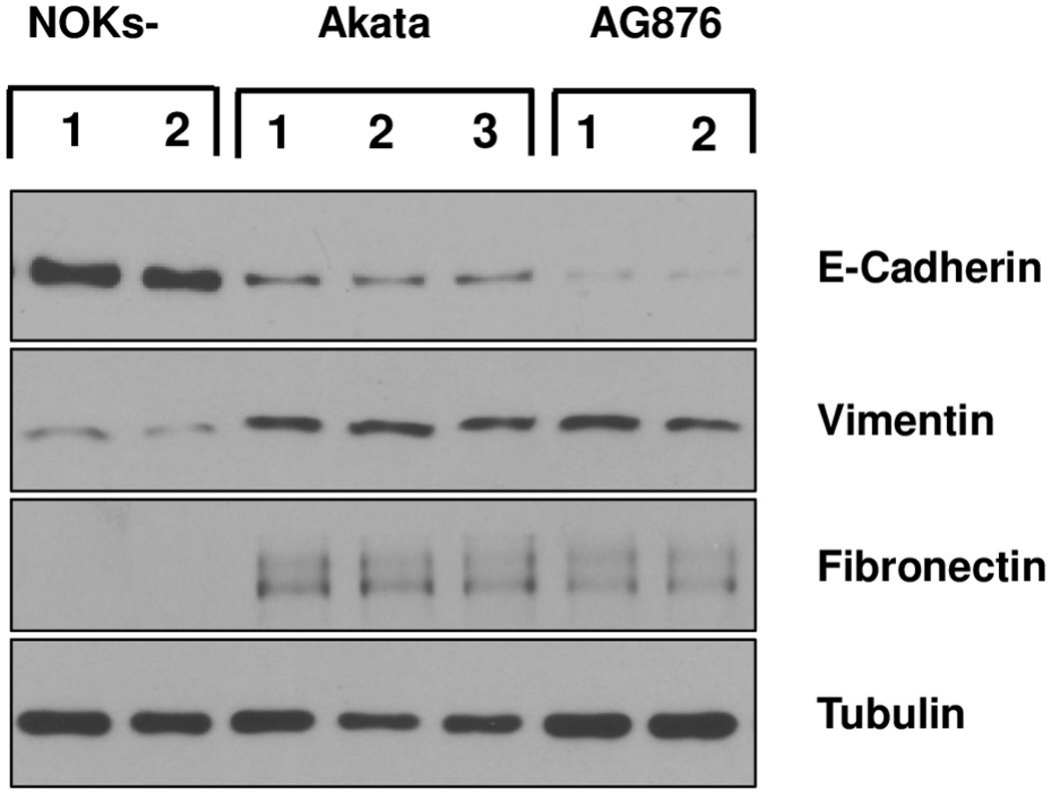
Type 1 and Type 2 EBV both induce EMT in NOKs. Uninfected, Akata EBV-infected, or AG876 EBV-infected NOKs (each in the context of the “NOKs-2 line”) were seeded in triplicate (125K cells per well) in 6 well plates and grown in KSFM medium without supplements for 24 hours. Immunoblot analysis was then performed to assess expression levels of E-cadherin, Vimentin, Fibronectin or tubulin (loading control).

Because the RNA-seq GSEA analysis showed that the “ONDER_CDH1_TARGETS_3_DN” gene set is down-regulated in both the AG876 virus-infected and Akata virus-infected NOKs in comparison to uninfected cells, we examined whether expression of the CDH1 gene product, e-cadherin, is reduced in NOKs infected with either the Akata or AG876 viruses, and if so, whether loss of e-cadherin expression is associated with induction of the EMT phenotype. Although Akata virus infection of NOKs was previously shown to decrease e-cadherin expression and increase expression of EMT markers [36], whether AG876 virus infection has a similar effect is unknown. We confirmed by immunoblot analysis that both Akata virus-infected NOKs, and AG876 virus-infected NOKs, express less e-cadherin in comparison to uninfected NOKs (Fig 4). In addition, we found that both Akata virus-infected and AG876 virus-infected cells express higher levels of two different markers of EMT, vimentin and fibronectin, in comparison to the uninfected NOKs.

**Fig 5:**
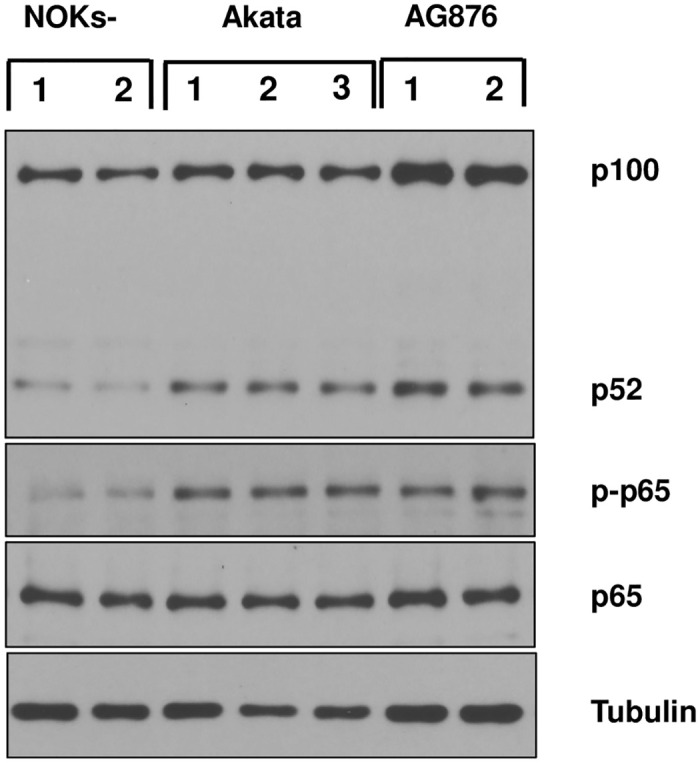
Type 1 and Type 2 EBV both induce NF-κB signaling in NOKs. Uninfected, Akata EBV-infected, or AG876 EBV-infected NOKs (each in the context of the “NOKs-2” line) were seeded in triplicate (125K cells per well) in 6 well plates and grown in KSFM medium without supplements for 24 hours. Immunoblot analysis was then performed to assess expression levels of p100/p52, total p65, phospho-p65, or tubulin (loading control). The same cellular extracts were used in this figure as in Fig 4.

As LMP1 is expressed in both the Akata virus-infected and AG876 virus-infected NOKs (Fig 3 and S6 Fig), and LMP1 can induce both the canonical and non-canonical NF-κB signaling pathways [39–41], we also performed immunoblot analysis to determine if either of these NF-κB pathways is activated in Akata virus-infected and/or AG876 virus-infected NOKs in comparison to uninfected NOKs. As shown in Fig 5, both Akata virus-infected cells and AG876 virus-infected cells have increased levels of the cleaved p52 protein (indicative of increased non-canonical NF-κB signaling) as well as increased levels of phosphorylated p65 protein (indicative of increased canonical NF-κB signaling).

### Uninfected NOKs spontaneously differentiate when cultured in growth factor-limiting conditions, and both type 1 Akata virus and type 2 AG876 virus infection inhibit this spontaneous differentiation

**Fig 6:**
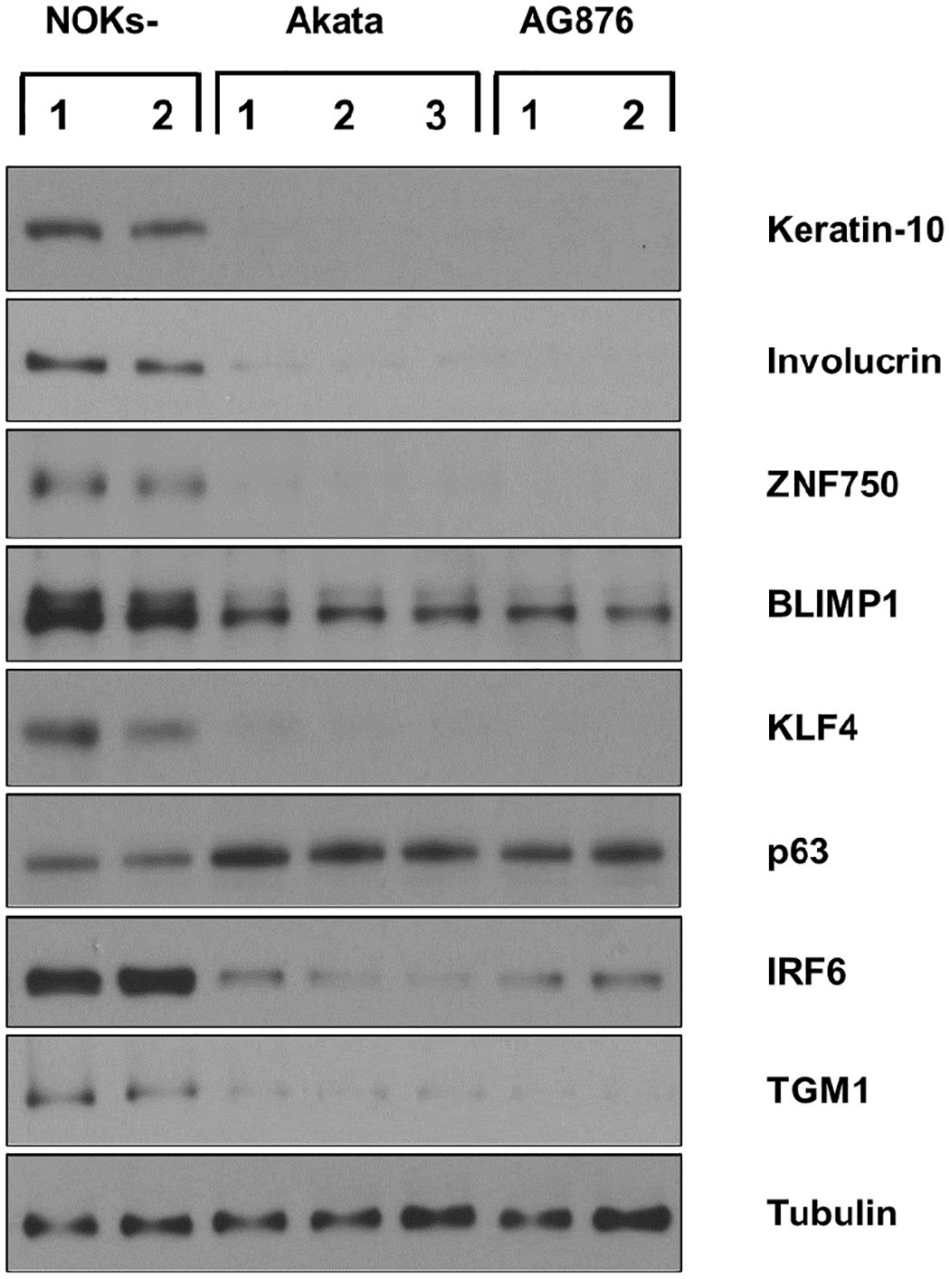
Type 1 and Type 2 EBV both inhibit spontaneous NOKs differentiation when growth factors are limiting. Uninfected, Akata EBV-infected, or AG876 EBV-infected NOKs (each in the context of the “NOKs-2 line”) were seeded in triplicate (125K cells per well) in 6 well plates and grown in KSFM medium without supplements for 24 hours. Immunoblot analysis was then performed to assess expression levels of Keratin-10 (K-10), Involucrin, ZNF750, BLIMP1, KLF4, delta p63, IRF6, TGM1 or tubulin (loading control) as indicated.

To confirm that both type 1 and type 2 EBV infection impede the ability of NOKs to spontaneously differentiate in the absence of EGF and BPE, we examined the expression levels of a variety of different epithelial cell differentiation markers in uninfected versus Akata virus- or AG876 virus-infected NOKs cells. As shown in Fig 6, uninfected NOKs grown in the absence of EGF and BPE express many different differentiation markers, including Keratin 10 (K10), involucrin, ZNF750, BLIMP1, KLF4, IRF6 and TGM1, and the expression of these differentiation markers is much decreased in both the Akata virus-infected and AG876 virus-infected NOKs. In contrast, uninfected NOKs express a lower level of the delta isoform of p63, a protein that is known to have decreased expression in differentiated epithelial cells [42,43]. As a control, we also created a NOKs line that is stably infected with an oriP-based vector expressing both the GFP and G418R genes (pDAO83, a gift from the Kathleen Burns laboratory via addgene) and found this line is similar to the uninfected NOKs in its ability to differentiate (S8 Fig). Of note, although RNA-seq analysis results (which included one sample of uninfected “NOKs-2” cells and two samples of uninfected “NOKs-1” cells), suggested that the uninfected “NOKs-2” cells have a higher level of spontaneous differentiation compared to the uninfected “NOKs-1” cells (S1 Table), and this difference was also seen by immunoblot analysis of differentiation-induced cellular proteins (S7 Fig), EBV infection of either NOKs line produced a similar decrease in differentiation (Fig 6 and S7 Fig). These results confirm that uninfected NOKs stop proliferating, and spontaneously differentiate, when grown in monolayer cultures at sub-confluent conditions in the absence of EGF and BPE and demonstrate that both type 1 and type 2 EBV infection promote proliferation, and inhibit differentiation, under these growth factor-limited conditions.

### Type 2 AG876 virus infection and Type 1 Akata virus infection also both inhibit epithelial cell differentiation induced by methylcellulose suspension

**Fig 7:**
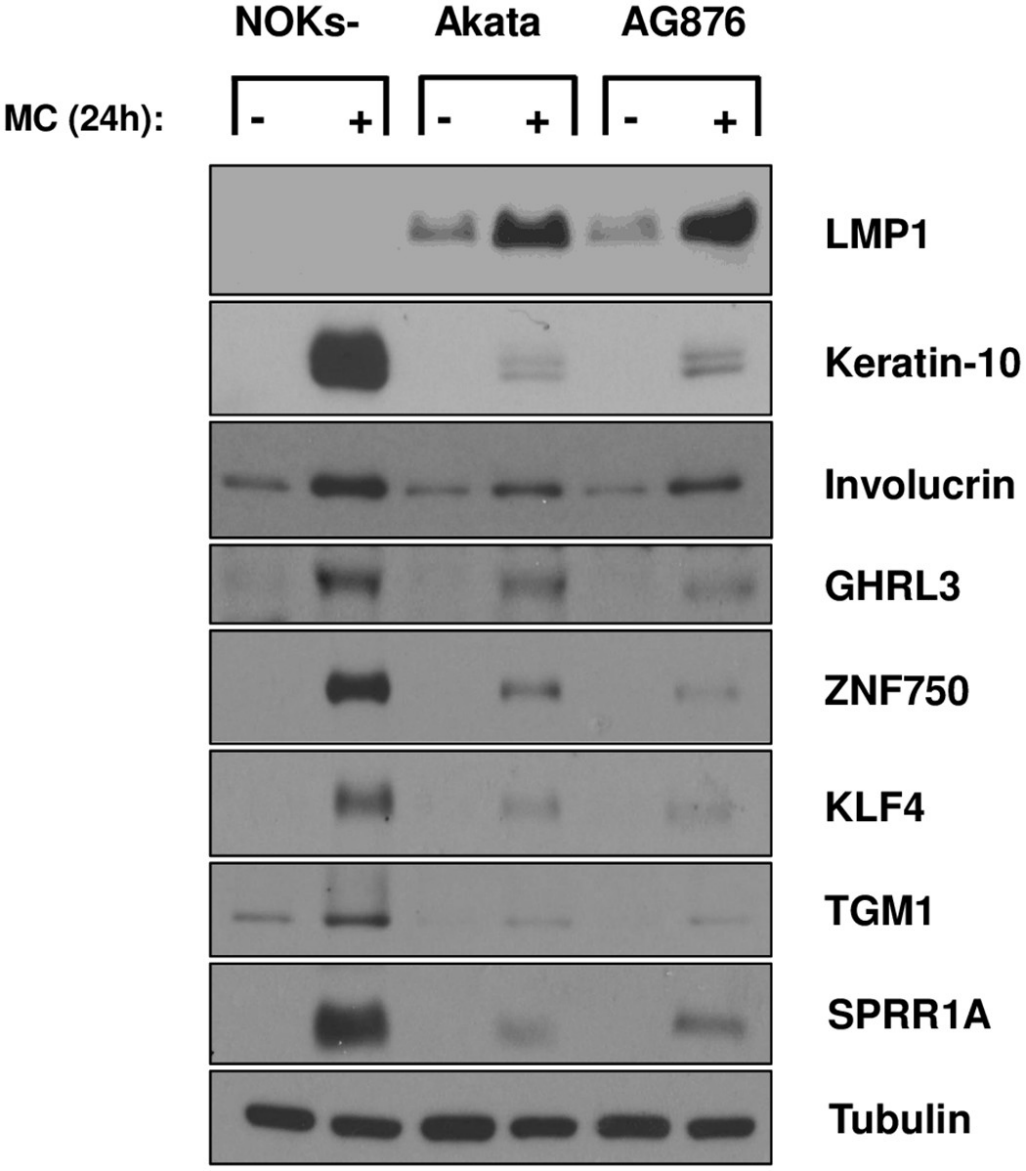
Type 1 and Type 2 EBV both inhibit methylcellulose-induced NOKs differentiation. Uninfected, Akata EBV-infected, or AG876 EBV-infected NOKs (each in the context of the “NOKs-2” line) were differentiated in 1.6% methylcellulose containing KSFM medium without supplements for 24 hours. Following differentiation, the cells were harvested and immunoblot analysis was then performed to assess expression levels of LMP1, K-10, Involucrin, GHRL3, ZNF750, KLF4, TGM1, SPRR1A and tubulin (loading control) as indicated.

To further compare the abilities of T1 Akata virus and T2 AG876 virus to inhibit NOKs differentiation, AG876 virus-infected and Akata virus-infected NOKs were suspended in methylcellulose as previously described by our group [13] for 24 hours, and then immunoblots were performed to compare the expression levels of various different epithelial cell differentiation markers. As shown in Fig 7, both Akata virus infection and AG876 virus infection decrease the expression levels of numerous different differentiation markers induced by methylcellulose suspension in uninfected NOKs, including K10, involucrin, GHRL3, ZNF750, KLF4, TGM1 and SPRR1A. As previously described by our group [13], LMP1 expression is increased by methylcellulose suspension in Akata virus-infected NOKs, and LMP1 is similarly increased by methylcellulose suspension in AG876 virus-infected NOKs.

### Type 2 AG876 virus infection and Type 1 Akata virus infection both induce invasion of the collagen matrix, and inhibit epithelial cell differentiation, when grown in air-liquid interface cultures

**Fig 8:**
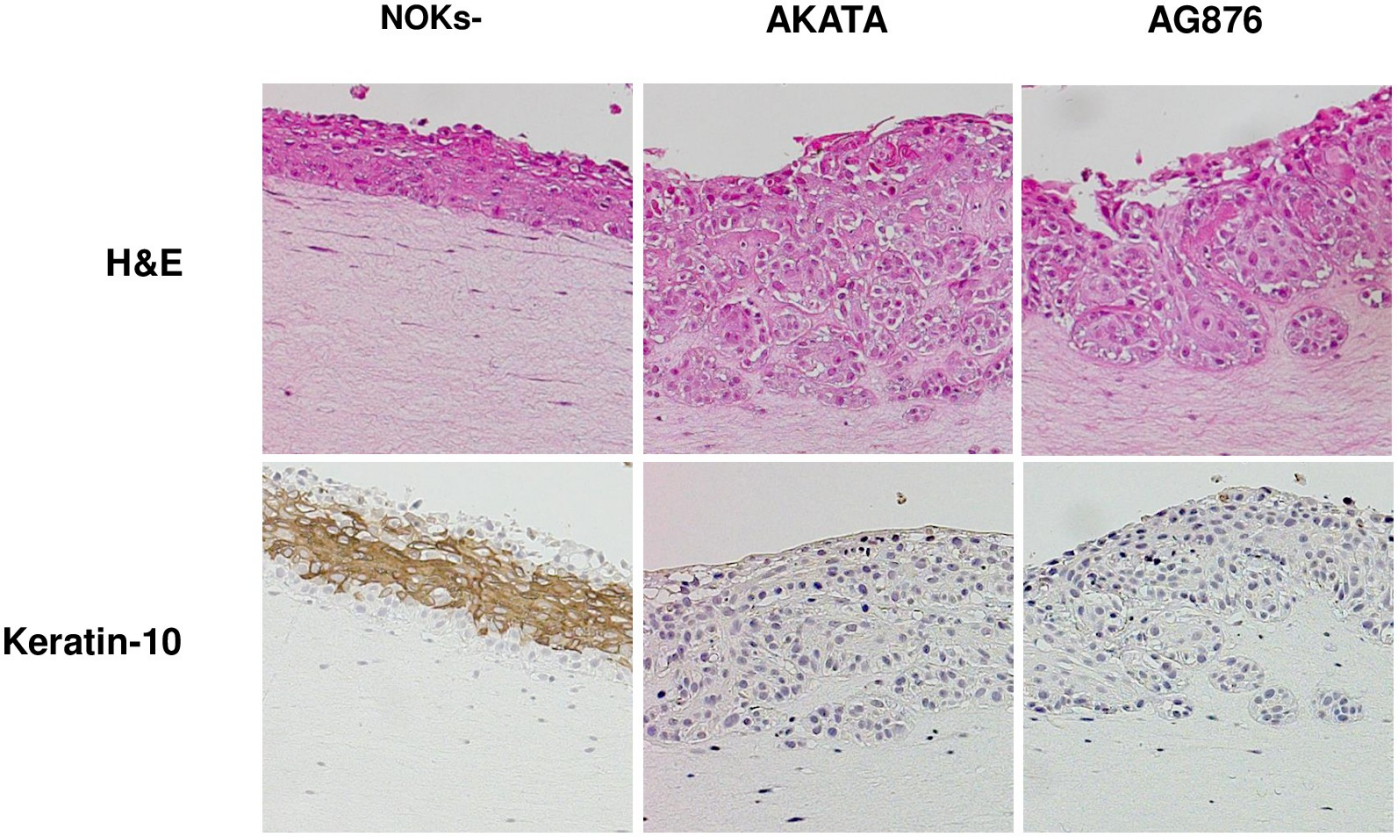
Type 1 and type 2 EBV both inhibit NOKs differentiation during rafting. Two conditions each of uninfected NOKs, Akata EBV-infected NOKs, and AG876 EBV-infected NOKs (each in the context of the “NOKs 2” line) were differentiated on raft cultures as described in the methods. Histology was performed on rafted cells and sections were stained for hematoxylin and eosin (H&E) or stained with anti-K-10 antibody and counterstained with hematoxylin. Representative sections are shown for uninfected NOKs and NOKs infected with Akata EBV or AG876 EBV.

We also compared the ability of Akata virus infection, versus AG876 virus infection, to decrease expression of the differentiation marker K10, and increase invasion of NOKs cells into the underlying collagen matrix, when cells were grown in air-liquid interface cultures (Fig 8). As previously described by our group [34], cells in the basal layer of Akata virus-infected NOKs invade the underlying collagen matrix when grown in “rafted” cultures, in contrast to the basal cells of the uninfected NOKs. Similar to the Akata virus infected cells, the AG876 virus-infected cells also invade the underlying collagen matrix (Fig 8). This result is consistent with the RNA-seq GSEA analysis showing that the “JAEGER_METASTASIS_DN” gene set is decreased in both Akata virus- and AG876 virus- infected NOKs (Fig 1), and the EMT phenotype observed in monolayer cultures of the Akata and AG876 virus- infected NOKs (Fig 4).

### Akata virus-infected and AG876 virus-infected NOKs have similar levels of lytic virus infection

**Fig 9:**
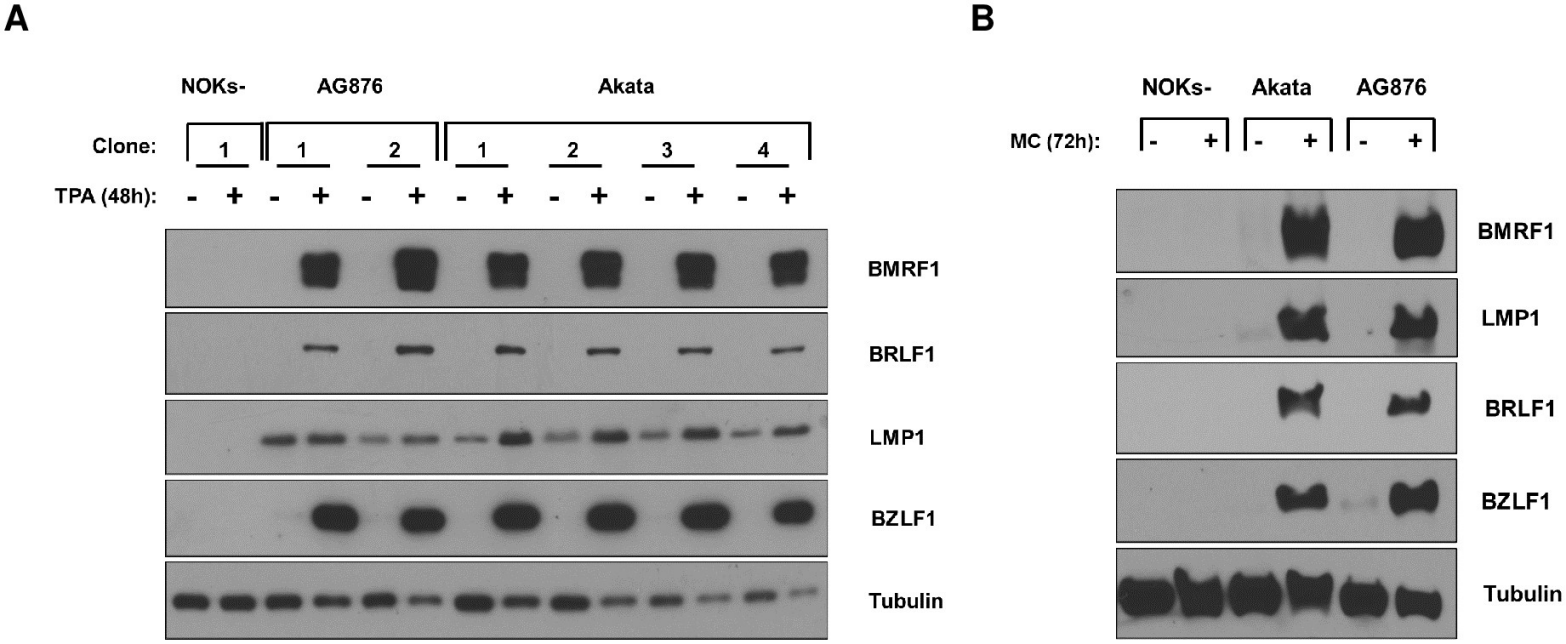
NOKs infected with Type 1 and type 2 EBV exhibit similar levels of lytic reactivation in response to differentiation stimuli. (A) Uninfected NOKs, two different lines of AG876 EBV-infected NOKs cells, and four different lines of Akata EBV-infected NOKs (each in the context of the “NOKs-2” line) were grown in sub-confluent conditions in KSFM medium without supplements for 24 hours. Cells were then treated with TPA at 20ng/mL for 48 hours, and immunoblot analysis was performed to assess expression of BZLF1, BRLF1, LMP1, BMRF1 or tubulin (loading control) as indicated. (B) Akata EBV-infected, AG876-EBV infected or uninfected NOKs were differentiated by suspending the cells in 1.6% methylcellulose containing KSFM medium without supplements for 72 hours, and then immunoblot analysis was performed to assess expression of the BZLF1, BRLF1, LMP1, BMRF1 and tubulin (loading control) proteins.

**Fig 10:**
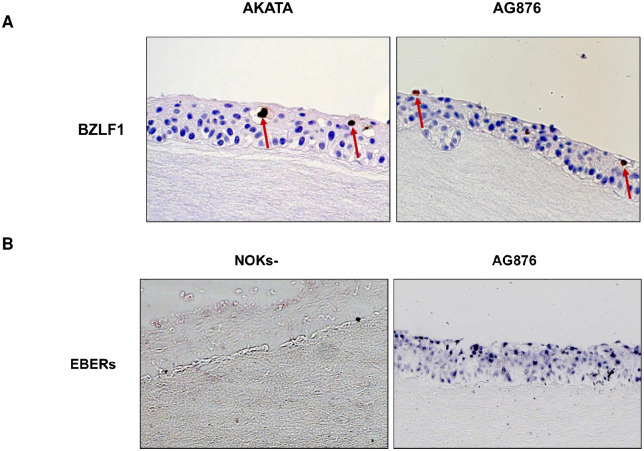
Rafted Type 1 and Type 2 EBV infected NOKs have similar levels of lytic EBV reactivation. Two lines each of Akata EBV-infected or AG876 EBV-infected cells were differentiated on raft cultures (each in the context of the “NOKS-2 line”). (**A**) Histology was performed on rafted cells and sections were stained with BZLF1 antibody and counter-stained with hematoxylin (choosing regions of the raft that had some level of K10 expression). Representative sections are shown for NOKs infected with Akata EBV or AG876 EBV. BZLF1-expressing cells are indicated by arrows. (**B**) In Situ hybridization was performed to detect EBV EBERs. Representative sections are shown for uninfected NOKs or NOKs infected with AG876 EBV.

The EBV transcript analysis of the RNA-seq results does not suggest that AG876 virus-infected and Akata virus-infected NOKs are significantly different in their ability to spontaneously lytically reactivate. However, given our recent finding that T2 EBV is more lytic than T1 EBV in lymphoblastoid B cell lines and in lymphomas of humanized mice [27,29], we explored whether T1 Akata virus-infected versus T2 AG876 virus-infected NOKs differ in their ability to switch to the lytic form of viral reactivation when treated with the phorbol ester TPA, suspended in methylcellulose, or rafted. The Kenney and Mertz labs have previously shown that the ability of TPA to induce lytic EBV reactivation of Akata virus-infected NOKs is at least partially dependent upon differentiation activated transcription factors such as BLIMP1 and KLF4 [12,44]. As shown in Fig 9A and 9B, AG876 virus-infected NOKs are similar to Akata virus-infected NOKs in their ability to lytically reactivate following TPA treatment (Fig 9A) or when suspended in methylcellulose (Fig 9B). Furthermore, both Akata virus-infected and AG876 virus-infected NOKs express similar levels of the IE BZLF1 protein in the differentiated upper cell layers in rafted cultures (Fig 10A). Similar to our previous results showing that EBERs are expressed in both undifferentiated and differentiated layers of rafted Akata virus-infected NOKs [12,13], EBERs were likewise expressed both undifferentiated and differentiated layers of rafted AG876 virus-infected NOKs (Fig 10B). These results suggest that NOKs infected with T1 Akata virus and T2 AG876 virus are similar in their ability to lytically reactivate in response to differentiating signals.

## Discussion

The ability of EBV to inhibit differentiation and promote proliferation of normal oropharyngeal cells is likely to be a major mechanism by which EBV induces undifferentiated NPC tumors in humans. Although we previously showed that latent type 1 EBV infection (Akata virus) inhibits the ability of NOKs to differentiate when “rafted” [33], whether type 2 EBV infection differs from type 1 EBV in epithelial cells has not been previously examined. In this study, we show that both type 1 and type 2 EBV enhance cellular proliferation and inhibit spontaneous differentiation in NOKs, and demonstrate that the phenotypic differences between EBV-infected and uninfected NOKs are most dramatic when growth factors are limiting. Furthermore, in contrast to our recent studies in B cells, we demonstrate that type 2 and type 1 EBV are similar in their ability to lytically reactivate in response to differentiation stimuli in NOKs. Thus, differences between type 1 and type 2 EBV infection appear to be cell type dependent.

Although type 1 EBV infection is found much more commonly in NPC tumors than type 2 EBV, the finding that at least some NPC tumors contain only type 2 EBV [19,45] suggests that the propensity for type 1 EBV infection in human NPCs is more likely due to the much greater frequency of type 1 versus type 2 EBV infection in regions of the world that have a high frequency of NPC tumors [19], rather than an inherent decrease in the ability of type 2 EBV to promote NPC. Consistent with this interpretation, we show here that type 1 and type 2 EBV infection produce similar proliferation and differentiation phenotypes in NOKs when growth factors are limiting.

Because EBV infection does not transform NOKs cells *in vitro*, and EBV genomes are not stably retained in infected cells unless the virus provides a survival advantage to cells [46,47], we first needed to insert an antibiotic selection marker into the AG876 virus genome to study its phenotype in NOKs. To construct a G418R/GFP+ AG876 virus that is similar to the G418R/GFP+ type 1 Akata virus (from the BX1 BL line) [37] previously used by the Kenney lab to study EBV-infected NOKs, we PCR-amplified the GFP/G418R cassette from the BXLF1 gene locus of the GFP-BXLF1 Akata virus (constructed by the Hutt-Fletcher lab [37]) and then inserted this cassette by homologous recombination into the same site (BXLF1 gene) of the AG876 genome. Thus, any differences between cells infected with the AG876 and Akata viruses in this or future studies are unlikely due to unintended effects of different viral genome insertion sites of the GFP/G418R cassette. In contrast, we previously found that another G418R/GFP expressing Akata bacmid construct (containing the GFP/G418R cassette inserted near the right hand oriLyt) unintentionally results in decreased expression of the viral BARTs microRNAs [34].

Although the precise viral RNAs/protein(s) responsible for the ability of EBV to induce proliferation and inhibit differentiation of NOKs are currently being identified by our laboratories, the most likely candidates are the two EBV latency proteins, LMP1 and LMP2A. Interestingly, EBV B95.8 strain LMP1 protein, but not CAO EBV strain LMP1 protein, was previously shown to inhibit differentiation when over-expressed in rafted SCC12F squamous carcinoma cells [48]. In contrast, B95.8 LMP1 and CAO LMP1 induced similar levels of NF-KB activity in SCC12F cells [48]. B95.8 EBV is a type 1 EBV strain derived from a mononucleosis patient in the USA, while CAO is a type 1 EBV strain derived from an Asian NPC tumor. The LMP1 sequences of the Akata, AG876, B95.8, CAO and M81 (derived from an NPC tumor in Asia) EBV strains are shown in S9 Fig. In addition to LMP1, the EBV LMP2A protein has also been reported to inhibit differentiation of rafted HaCAT cells when over-expressed [49], and to increase expression of the delta p63 protein [50]. Further studies will be required to determine if one or both latent EBV membrane proteins contribute to the ability of EBV to inhibit epithelial cell differentiation in NOKs, and if LMP1 strain variations alter EBV’s ability to inhibit differentiation, particularly when LMP1 is expressed at biologically relevant levels and in the context of the intact viral genome. Of note, our labs previously showed that an EBV Akata bacmid that loses expression of the BARTs viral microRNAs retains the ability to inhibit differentiation of rafted NOKs [34].

NPC tumors contain largely latent EBV infection, and excessive lytic infection induced by either type 1 or type 2 EBV infection in undifferentiated epithelial cells would be expected to inhibit the development of NPC. We find here that both type 1 and type 2 viruses produce latent EBV infection in the undifferentiated basal layers of rafted NOKs and undergo similar amounts of lytic reactivation in response to the phorbol ester TPA, or differentiation induced by suspension in methylcellulose or rafting in air-liquid interface culture. As infectious EBV in the saliva is largely derived from lytically infected oropharyngeal cells [5] and is required for spread of the virus from host to host, these results also suggest that type 1 and type 2 EBV may be similarly efficient in the ability to infect new hosts. However, additional factors (such as the ability to evade the host immune response) will also determine the infectivity of each virus type and need to be further investigated.

Our finding here that the lytic reactivation, proliferation and differentiation phenotypes of type 1 and type 2 EBV-infected NOKs are similar, in contrast to our previous findings in newly infected B cells [29], are not particularly surprising given that the most divergent genes in type 1 versus type 2 EBV (EBNA2 and EBNA3A/B/C) are not generally expressed in EBV-infected epithelial cells. Latently EBV-infected epithelial cells (which may be restricted to tumor cells in humans) have “type I” or “type II” latent infection, in which at most three latent viral proteins (EBNA1, LMP1 and LMP2A) are expressed, along with small viral nuclear RNAs and virally-encoded microRNAs [4,11]. In addition to the lack of EBNA2 and EBNA3A/B/C expression in EBV-infected epithelial cells, epithelial cells do not express the B-cell receptor (BCR), and we previously found that enhanced BCR activity is a major contributor to the increased lytic EBV infection that occurs in B cells with type 2 EBV infection [27,29]. In contrast to BCR-mediated lytic EBV reactivation in B cells, our previous studies have suggested that lytic EBV reactivation in epithelial cells is largely mediated by epithelial cell differentiation signals [12,13]. Interestingly, we also recently showed that the hippo signaling effectors, YAP and TAZ, can also induce lytic EBV reactivation in a differentiation-independent manner in epithelial cells by activating the BZLF1 promoter in conjunction with TEAD family members [51]. However, since the type 1 and type 2 EBV viruses in this study responded similarly to TPA in NOKs cells (which requires YAP/TAZ to efficiently induce lytic EBV reactivation in NOKs cells [51]), type 1 and type 2 EBV are likely to also respond similarly to YAP/TAZ mediated lytic EBV reactivation in NOKs.

It is currently unclear whether NFATc1, NFATc2 or other NFAT family members can contribute to lytic EBV reactivation in epithelial cells, similar to their effects in EBV-infected B cells. Although RNA-seq analysis of EBV infected and uninfected NOKs showed minimal expression of either the NFATc1 or NFATc2 transcripts (S1 Table), there is detectable expression of the NFATc3 and NFAT5 transcripts. Nevertheless, NFATc1 has been reported to increase differentiation of keratinocytes, and immunosuppressant calcineurin inhibitor drugs such as cyclosporin have been proposed to induce squamous cell carcinomas in patients at least partially via their inhibitory effects on NFATc1 [52,53]. Since both the type 1 Akata virus and type 2 AG876 viruses used in our experiments have the NFAT-responsive Zp-V3 form of the BZLF1 IE promoter, the two viruses may respond similarly to the effects of NFAT family members in epithelial cells. Future studies should address whether type 1 viruses containing the Zp-P form of the BZLF1 promoter respond differently compared to type 1 viruses with the Zp-V3 form of the BZLF1 promoter when exposed to differentiating agents and/or NFAT inhibitors in epithelial cells.

In summary, we show here that both type 1 and type 2 EBV infection induce cellular proliferation, and inhibit differentiation, of NOKs, particularly when growth factors are limiting. Together, these results suggest that the EBV-infected NOKs are an excellent model for dissecting mechanism(s) by which EBV infection promotes NPC. Finally, our finding that type 1 and type 2 EBV infection of NOKs have a similar lytic phenotype suggests that differences between type 1 and type 2 EBV infection are likely cell-type dependent, with some cell types such as B cells showing more distinct type-specific phenotypes compared to other cell types such as epithelial cells.

## Materials and methods

### Cell lines and cell culture

The normal oral keratinocytes (NOKs) cell line (a generous gift from Karl Munger of Tufts University (via Paul Lambert and Bill Sugden of the University of Wisconsin) is a telomerase-immortalized normal oral keratinocyte cell line, grown in keratinocyte serum-free media supplemented with 12.5 mg bovine pituitary extract, and 0.1 μg epidermal growth factor per 500 ml of media (KSFM, Lifetech). NOKs were derived as previously described [33]. The Burkitt lymphoma cell line BX1 (a gift from Lindsay Hutt-Fletcher) was derived as previously described by super-infecting an EBV-negative Akata Burkitt lymphoma cell clone with the Akata strain of EBV (containing a G418 resistance gene cassette and GFP gene inserted into the EBV BXLF1 gene) [37] and was maintained with RPMI media with 10% fetal bovine serum with 1% pen-strep and 500 μg/ml G418 antibiotic selection. EBV-infected NOKs-Akata cells were created as previously described [54], except that uninfected NOKs were co-cultured with lytically induced BX1 BL cells as the source of Akata virus infection. NOKs Akata cells and NOKs-AG876 cells were maintained with 50 μg/ml G418 antibiotic selection in addition to the media/growth supplements used to grow NOKs. EBV-negative Akata cells were a kind gift from Kenzo Takada of Hokkaido University, Japan, via Bill Sugden of the University of Wisconsin and have been previously derived as described [55]. AG876, originally derived by Pizzo et al. [56], is a Burkitt lymphoma cell line containing T2 EBV and were obtained as gift from Dr. Bill Sugden at the University of Wisconsin-Madison. EBV-negative Akata Burkitt lymphoma cells and EBV-positive AG876 Burkitt lymphoma cells were maintained in RPMI media with 10% fetal bovine serum with 1% pen-strep.

### Construction of AG876-GFP virus

The GFP/G418R cassette (and the surrounding EBV BXLF1 gene sequences) that was previously inserted within the Akata virus BXLF1 (TK) gene in Akata BX1 cells [37]) was PCR-amplified using two different primer sets. The BXLF1-GFP portion was amplified using the primers 5’-CCGCTCTAGAACTAGTGGATC*ATTTAAAT***CAGGCAGGGGAATTCAGG-3’** and 5’-GCTTCTCCTATAGTG-3’ (amplifying the 5’ region of the GFP/G418R cassette) and the 3” region of the cassette was amplified using the primers 5’-GTATCCATCATGGCTGATGCAATGCGGCGG-3’ and 5’-CCAGGGCCCCCCCTCGAGGTCG*ATTTAAAT***GCCCGCCCGGCGGCTGGCGAAAATGTCAGG** (standard text anneals to pBKS-, italicized incorporates a SwaI site, and the bold anneals to BXLF1). The PCR products containing the GFP/G418R gene cassette along with 784 bp of BXLF1 on the 5’ side (basepairs 130,658–131,442 of Genbank Accession #LN827548.2) and 1364 bp on the 3’ side (basepairs 131,449–132,813) of the GFP/G418R cassette with flanking SwaI sites, were then cloned into pBKS vector (Agilent, Santa Clara CA,USA) using the Gibson assembly method (NEB Builder kit, New England Biolabs) to reassemble the complete GFP/G418R cassette along with the adjacent EBV BXLF1 gene sequences on either side, and sequenced to confirm its correct identity. The DNA fragment containing the GFP/G418R gene cassette along with of the adjacent Akata virus BXLF1 gene sequences on either side of the GFP/G418R cassette was then isolated from the plasmid by SwaI digest and 1 ug of this DNA was electroporated into AG876 Burkitt lymphoma cells using the Amaxa Nucleofector 2b device (Lonza, Morristown, NJ) with program A30, Buffer V. G418-resistant AG876 BL cells were then selected using G418 at 500ug/ml.

### Creation of AG876-GFP infected HeLa cell clones and AG876-GFP infected Burkitt cell clones

G418R AG876 BL cells (created as described above) were lytically reactivated by treating cells with 3 mM sodium butyrate and 20ng/ml TPA overnight. After removing the TPA and sodium butyrate by washing cells in PBS, BL cells were co-cultured with HeLa cells for 48 hours and then removed. HeLa cell clones infected with the AG876-GFP virus were selected by treating cells with 500ug/ml G418. Infectious AG876-GFP virus was then produced from the infected HeLa cells (grown in RPMI media) by transfecting the cells with BZLF1 and BRLF1 expression vectors. Two days after transfection, media from the transfected HeLa cells was added to EBV-negative Akata BL cells. After another 2 days, the BL cells were selected for infection with the AG876-GFP virus by adding G418 (500 ug/ml) to the media.

### Creation of AG876-GFP infected NOKs lines

Once stably AG876-GFP infected BL cells were obtained, virus was lytically reactivated by treating the cells with TPA and sodium butyrate for 18 hours, and then after removing the RPMI media (along with the TPA and sodium butyrate) the BL cells were resuspended in KFSM media and co-cultured with uninfected NOKs for several days. After washing off the BL cells, NOKs lines were then selected for stable AG876-GFP infection by treating the cells with G418 (50 ug/ml). Two different uninfected NOKs lines (“NOKs-1” and “NOKs-2”) that had been separately maintained in two different laboratories were each infected with both virus types; similar results were obtained in each of the two NOKs lines.

### Assessing Proliferation of uninfected versus Akata EBV-infected or AG876 EBV-infected NOKs in monolayer cultures under growth-factor restricted conditions

50,000 cells of uninfected NOKs, Akata EBV-infected NOKs, or AG876 EBV-infected NOKs were uniformly seeded into each well of a 6 well plate, and then grown in KSFM medium without any EGF or BPE supplement. Uninfected NOKs were seeded in triplicate, while three different lines of Akata EBV-infected NOKs or AG876 EBV-infected NOKs were used. The cells were allowed to grow for 5 days, and then counted using trypan blue staining. The number of cells obtained from Akata EBV-infected or AG876 EBV-infected NOKs was normalized to the number of cells obtained from the uninfected NOKs (set as 1).

### Assessing the phenotypes of uninfected versus Akata EBV-infected or AG876 EBV- infected NOKs in monolayer cultures using immunoblot analyses

125K cells were uniformly seeded in each well of a 6W plate in KSFM medium without supplements. Cell extracts were collected 24 hours later for immunoblot analysis. Immunoblots were performed as previously described [57]. Cells were lysed in lysis buffer (1:3 mixture of buffer I (5% sodium dodecyl sulfate (SDS), 0.15 M Tris-HCl (pH 6.8), 30% glycerol) and buffer II (25 mM Tris-HCl (pH 8.3), 50 mM NaCl, 0.5% NP-40, 0.5% deoxycholate, 0.1% SDS)) with protease inhibitors (cOmplete, Roche), and then quantitation of protein was performed using the DC Bio-rad protein assay. Proteins were run on a 10% polyacrylamide gel and then transferred onto a nitrocellulose membrane. The membranes were blocked with 1X PBS mixed with 5% milk and 0.1% Tween 20 for one hour and then incubated with primary antibody overnight. Membranes were washed with wash buffer (1X PBS, .1% Tween 20) three times for 5 minutes, and then incubated with secondary antibody suspended in 5% milk for one hour, before washing with wash buffer three times for 10 minutes. Membranes were then treated with ECL (Thermo Fisher, Waltham, MA) and imaged.

### Immunohistochemistry (IHC) and EBERs studies

Paraffin-embedded slides were heated on 70°C heat blocks, deparaffinized in xylene, and then hydrated in a series of alcohols (100%, 90%, 70%) for a period of 5 minutes each. After boiling slides in 10mM Tris, 1 mM EDTA (pH 9.0) for 20 minutes, slides were blocked with 2.5% horse serum and primary antibodies were added to the slides overnight at 4°C. After washing in 1X PBS, secondary antibody was added (Impress secondary antibody, Vector Labs, Burlingame, CA) for thirty minutes. DAB (Cell Signaling, Danvers, MA) was added for a period of approximately 1 minute, Hematoxylin was used as a counterstain and the slides were dehydrated in alcohols before mounting. Anti-BZLF1 antibody (BZ.1 clone, Santa Cruz SC53904) was used at a 1:200 dilution, K10 antibody (Biolegend catalogue #905404) was used at 1:4000. All dilutions were in 2.5% Normal Horse Serum (Vector Labs, Burlingame, CA). EBER *in situ* hybridization studies were performed using the PNA ISH Detection Kit (DakoCytomation) according to the manufacturer’s protocol as previously described [58].

### Chemicals

Phorbol 12-myristate 13-acetate (TPA) was purchased from Sigma (catalog #P8139) and used at 20 ng/ml (diluted in DMSO). Sodium Butyrate was purchased from Sigma (Catalog #B5887) and used at 3mM. Control conditions were treated equal amounts of the solvent.

### Antibodies used for immunoblots

The following antibodies were used for immunoblot analyses in this study: anti-PCNA (Cell Signaling Technologies, catalog #13110S, dilution 1: 1000), anti-p21 (Santa Cruz Biotechnologies, catalog #sc-56335, dilution 1: 500), anti-Keratin-10 (Biolegend, catalog# 905404, dilution 1:10000), anti-Involucrin (Sigma, catalog# I9018, dilution 1:5000), anti-ZNF750 (Sigma, catalog# HPA023012, dilution 1:500), anti-BLIMP1 (Cell Signaling Technologies, catalog #9115S, dilution 1:1000), anti-KLF4 (Cell Signaling Technologies, catalog # 12173S, dilution 1:1000), anti-p63 (Cell Signaling Technologies, catalog # 13109S, dilution 1:1000), anti-IRF6 (Biolegend, catalog #674502, dilution 1:1000), anti-TGM1(Novus, catalog # NBP2-34062, dilution 1:2000), anti-Tubulin (Sigma, catalog # T5168, dilution 1:5000), anti-Vimentin (Santa Cruz, catalog # sc-6260, dilution 1:500), anti-Fibronectin (Santa Cruz, catalog # sc-8422, dilution 1:500), anti-p65 (Cell Signaling Technologies, catalog # 4764S, dilution 1:1000), anti-phospho p65 (Cell Signaling Technologies, catalog # 3033S, dilution 1:1000), anti-p100/p52 (Cell Signaling Technologies, catalog # 3017S, dilution 1:1000), anti-E-cadherin (Cell Signaling Technologies, catalog # 3195S, dilution 1:1000), anti-GHRL3 (Novus, catalog # NBP1-80356, dilution 1:500), anti-SPRR1A (Abclonal, catalog # A17535, dilution 1:20000), anti-BZLF1 (Santa Cruz, catalog # sc-53904, dilution 1:500), anti-BMRF1 (Millipore, catalog # MAB8186, dilution 1: 2500), anti LMP2A (Santa Cruz, catalog #sc-101314, dilution 1:500), anti-EBNA1 (Santa Cruz, Catalog #sc-57719, dilution 1:500), anti-EBNA2 (Abcam, catalog #ab90543, dilution 1:1000) and anti-LMP1 (Abcam, Catalog # ab78113, dilution 1:1000). Anti-BRLF1 rabbit polyclonal antibody was directed against the R peptide (EDPDEETSQAVKALREMA), and was used at a dilution of 1: 2500. The secondary antibodies used were Horseradish peroxide (HRP)- labeled goat anti-mouse antibody (Thermo Scientific # G-21040, dilution 1:5000), and HRP- labeled goat anti-rabbit antibody (Thermo Fisher Scientific # G-21234, dilution 1:5000).

### Plasmids

All plasmid DNA was prepared using the Qiagen Maxi-prep kit according to the manufacturer’s instructions. The plasmid pSG5 was purchased from Stratagene. pSG5-R and pSG5-Z (kind gifts from Diane Hayward of John Hopkins University) contain the BZLF1 (Z) and BRLF1 (R) immediate-early genes driven by the SV40 promoter as previously described [59,60]. The plasmid pDA083 that was used to make the stable NOKs-GFP/G418R line was a gift from the Kathleen Burns laboratory via addgene.

### Organotypic rafting

Uninfected NOKs, Akata virus infected NOKs and AG876 virus infected NOKs were stratified by organotypic rafting as described previously [12]. Briefly, dermal equivalents were created using transwell inserts (24 mm diameter, 0.4 μM pore Costar) coated with a 1ml collagen mix (3 mg/ml Wako) containing F-media, 10% FBS and 1% pen-strep, followed by an additional 2.5 ml collagen mix containing F-12 media, 10% FBS, 1% pen-strep, and 4.5 X 10^5^ early- passage human fibroblasts (EF-1-F). The dermal equivalents were then suspended in F-12 medium with 10% FBS and 1% pen-strep. After four days, 2.1 x 10^5^ uninfected or EBV-infected NOKs cells were plated on the dermal equivalent and suspended in keratinocyte plating media (F-medium [1.88 mM Ca2^+^]) with 0.5% FBS, adenine (24 μg/ml), cholera toxin (8.4 ng/ml), hydrocortisone (2.4 μg/ml), and insulin (5 μg/ml). After another four days, the media was switched to cornification media (keratinocyte plating medium containing 5% FBS and 10 μM C_8:0_), and the cells were lifted to the air liquid interface. Cornification media was replaced every other day, and the cells were harvested after another 11 days. Cells were then embedded in 2% agar-1% formalin, fixed in 10% neutral buffered formalin overnight, and then embedded in paraffin and sectioned in 4 μM cross sections.

### Methylcellulose differentiation

1.6% methylcellulose (MC) was dissolved in KSFM without supplements and a homogeneous solution was prepared. 20 mL of MC solution was placed in 100 mm cell culture dishes and equilibrated at 37°C. One million NOKs, NOKs-Akata, or NOKs-AG876 cells were added into the MC solution-containing dishes and mixed well using a pipet tip. Following incubation of the cells in MC for 48 hours, cells were diluted in a 1:5 volume of phosphate-buffered saline (PBS), and then centrifuged and washed with additional PBS. The collected cells were then lysed in SDS lysis buffer for immunoblot analysis.

### RNA-seq analysis of T1 and T2 NOKs

RNA-seq libraries were prepared as previously described [61]. Briefly, uninfected, Akata EBV-infected or AG876 EBV-infected NOKs were grown in 0.2ng/ml EGF and 25ug/ml Bovine Pituitary Extract in KSFM (Thermo Fisher, Waltham, MA), then starved for 24 hours in KSFM without any supplements, then harvested in TRIzol (Thermo Fisher, Waltham, MA). RNA was isolated using the Direct-zol RNA MiniPrep Kit (Zymo Research, Irvin, CA) and RNA quality was assessed using an Agilent TapeStation. Ribodepleted library preparation using the Swift Rapid library prep kit, and sequencing on an Illumina NovaSeq 6000 with 50-bp paired-end reads, was performed by the Oklahoma Medical Research Foundation Clinical Genomics Center (Oklahoma City, OK). RNA-seq analysis of host transcription was conducted by BioInfoRx (Madison, WI) as previously described [61]. Briefly, fastQC was used to verify raw data quality of the Illumina reads, and then reads were aligned to the GRCh38 (hg38) human genome primary assembly using Subjunc aligner from Subread [62] and assigned to genes using Ensembl annotation (v93). Raw counts were normalized using the TMM normalization method [63] using edgeR and the normalized gene counts were transformed to log2 scale using the voom method from the R Limma package [64], then used for differential expression analysis. Functional interpretation of the differentially expressed genes was conducted based on GO terms, KEGG pathway and GSEA [65,66] methods.

### GSEA analysis

A ranked gene list was obtained from the differential gene expression analysis results according to the -log10(p value) multiplied by the sign of the log2(fold change). Molecular pathways and their corresponding gene sets were gathered from the Broad Institute of Molecule Signature Database (MSigDB; http://www.broadinstitute.org/gsea/msigdb) [67,68]. Gene set enrichment analysis (GSEA) was then performed using the fgsea package (1.22.0) [69] in R by providing the ranked gene list with the predefined MSigDB H, C2, C4, or C5 GO BP gene set collections (S4 Fig) or a manually combined collection of MSigDB gene sets (Fig 1). Enrichment plots were sorted by Normalized Enrichment Scores (NES) and filtered for Bejamini-Hochberg (BH)-adjusted p-value < 0.05 and plotted using the ggplot2 package (3.3.5) in R [70].

### EBV gene expression analysis

Reads were aligned to the GRCh38 (hg38) human genome primary assembly concatenated with Type 1 EBV genome (NC_007605.1) or the AG876 Type 2 genome (DQ279927.1) using STAR version 2.6.1a [71]. BAM files were converted into normalized bedGraph files for EBV regions using deeptools bamCoverage with the following parameters: -bs 1 -of bedgraph -region <EBV chromosome name>—normalizeUsing CPM [72]. The normalized bedGraph files were converted to variable-step wiggle format using the UCSC utilities bedGraphToBigWig and BigWigToWig [73]. Line plots displaying averaged wiggle data with standard error were generated using R [74] with genefilter [75]. Gene annotations were generated using the UCSC Genome Browser and Track Data Hubs for Type 1 EBV or AG876 with a bedfile annotation for the corresponding EBV genome [76,77].

## Supporting information

S1 Fig Cellular gene expression in Type 1 Akata EBV-infected NOKs versus Type 2 AG876 EBV-infected NOKs. The top 100 differentially expressed cellular genes in the RNA-seq analysis are shown. Names for each cell line, as well as the EBV type and strain are shown. Red indicates a gene is upregulated in corresponding cells and blue indicates it is down-regulated. EBV-infected cells were all derived from the “NOKs-1” line and the uninfected NOKs were a mixture of two NOKs-1 samples and one NOKs-2 sample as indicated in S1 Table.
(PDF)

S2 Fig Comparison of cellular transcripts in Type 2 AG876 EBV-infected NOKs versus uninfected NOKs. The top 100 differentially expressed cellular genes in the RNA-seq analysis are shown. Names for each cell line, as well as the EBV type and strain are shown. EBV-infected cells were all derived from the “NOKs-1” line and the uninfected NOKs were a mixture of two NOKs-1 samples and one NOKs-2 sample as indicated in S1 Table. Red indicates a gene is upregulated in corresponding cells and blue indicates it is down-regulated.
(PDF)

S3 Fig Cellular gene expression in Type 1 Akata EBV-infected NOKs versus uninfected NOKs. The top 100 differentially expressed cellular genes in the RNA-seq analysis are shown. Names for each cell line, as well as the EBV type and strain are shown. EBV-infected cells were all derived from the “NOKs-1” line and the uninfected NOKs were a mixture of two NOKs-1 samples and one NOKs-2 sample as indicated in S1 Table. Red indicates a gene is upregulated in corresponding cells and blue indicates it is down-regulated.
(PDF)

S4 Fig Gene set enrichment analysis (GSEA) suggests increased proliferation, decreased keratinocyte differentiation and decreased E-cadherin signaling in both the type 1 EBV-infected and type 2 EBV-infected NOKs in comparison to uninfected NOKs when growth factors are limiting. Results from GSEA for RNA-seq data of (**A, C, E, G**) Akata T1 or (**B, D, F, H**) AG876 T2 virus-infected NOKs versus uninfected NOKs as described in the Materials and Methods sections. Gene sets from the following MSigDB collections were analyzed: (**A-B**) hallmark, (**C-D**) curated, (**E-F**) computational, (**G-H**) and ontology sub-collection gene ontology biological process. Each enrichment plots display the top 10 up-regulated and top 10 down-regulated pathways with Benjamini-Hocheberg (BH)-adjusted p values of < 0.05 and were sorted by Normalized Enrichment Scores (NES). Pathways upregulated in the EBV-infected NOKs relative to uninfected NOKs are associated with NES values greater than 0, and down-regulated pathways are associated with NES values less than 0.
(TIF)

S5 Fig EBV gene expression in T2 virus- versus T1 virus-infected NOKs. RNA-seq reads from NOKs cells infected with T1 or T2 viruses were aligned to the T1 or T2 EBV genomes, respectively. For each strain, wiggle tracks of normalized read depth were normalized and plotted (black) with standard errors (gray). Annotation tracks for type 1 and type 2 viruses showing latent (blue) genes and lytic (black) genes were generated using UCSC genome browsers and displayed above. The BXLF1 gene (shown in green) was disrupted by insertion of a G418R/GFP cassette and transcription in this region arises as a result of the promoters within this cassette.
(TIF)

S6 Fig Comparison of latent EBV protein expression in uninfected, T2 AG876 virus-infected and T1 Akata virus-infected NOKs. The expression levels of LMP1, LMP2A, EBNA2, and EBNA1 in uninfected, Akata virus infected and AG876 virus infected NOKs cells (each in the context of the “NOKs 2” line) was examined by immunoblot analysis. LCL-AG876 was also included in each blot and served as a positive control for all the EBV latent proteins probed here. Actin was also measured as a loading control.
(TIF)

S7 Fig Type 1 and Type 2 EBV both inhibit spontaneous NOKs differentiation when growth factors are limiting. Uninfected NOKs (either the “NOKs-1” line or the “NOKs-2” line as indicated), or Akata EBV-infected, or AG876 EBV-infected NOKs (both in the context of the “NOKs-1” line) were seeded (125K cells per well) in 6 well plates and grown in KSFM medium without supplements for 24 hours. Immunoblot analysis was then performed to assess expression levels of LMP1, PCNA, Keratin-10 (K-10), Involucrin, BLIMP1, KLF4, delta p63, TGM1, SPRR1A or tubulin (loading control) as indicated.
(TIF)

S8 Fig Insertion of GFP/G418R does not affect the spontaneous differentiation of NOKs cells. Uninfected (“NOKs-2”), NOKs-2 cells stably transfected a vector expressing the GFP and G418 resistance genes (GFP/G418R), on NOKs-2 cells infected with Akata EBV or AG876 EBV were seeded (125K cells per well) in 6 well plates and grown in KSFM medium without supplements for 24 hours. Immunoblot analysis was then performed to assess expression levels of LMP1, PCNA, Keratin-10 (K-10), Involucrin, SPRR1A, TGM1, KLF4 or tubulin (loading control) as indicated.
(TIF)

S9 Fig Comparison of LMP1 protein sequences in different EBV strains. The LMP1 sequences of B95.8, CAO, AG876, M81, and Akata strains of EBV are compared. Amino acids that are different between strains are highlighted in yellow.
(TIF)

S1 Table Bulk RNA-seq data of Type 1 and Type 2 EBV-infected NOKs or uninfected NOKs cells. Uninfected and EBV infected NOKs cell samples are labelled to indicate whether they were derived from the “NOKs-1” line or the “NOKs-2” line.
(XLSX)
