# Supplementary figures and images for: Type 1 and Type 2 Epstein-Barr viruses induce proliferation, and inhibit differentiation, in infected telomerase-immortalized normal oral keratinocytes
Source: PLoS Pathog. 2022 Oct 3;18(10):e1010868. doi: 10.1371/journal.ppat.1010868 (PMC9529132; doi:10.1371/journal.ppat.1010868)

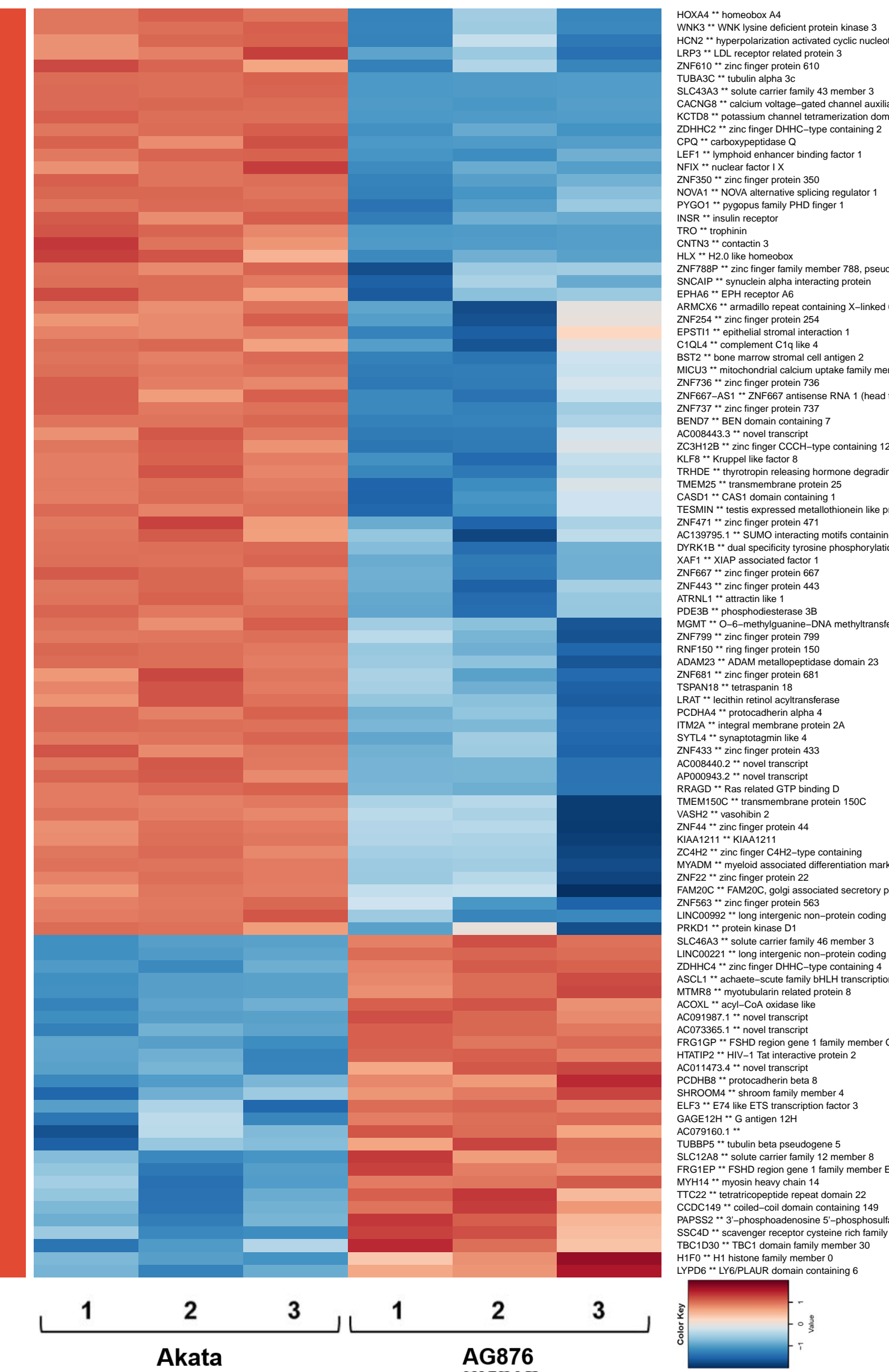

Supplement: S1 Fig — The top 100 differentially expressed cellular genes in the RNA-seq analysis are shown. Names for each cell line, as well as the EBV type and strain are shown. Red indicates a gene is upregulated in corresponding cells and blue indicates it is down-regulated. EBV-infected cells were all derived from the “NOKs-1” line and the uninfected NOKs were a mixture of two NOKs-1 samples and one NOKs-2 sample as indicated in S1 Table. (PDF) [file ppat.1010868.s001.pdf]

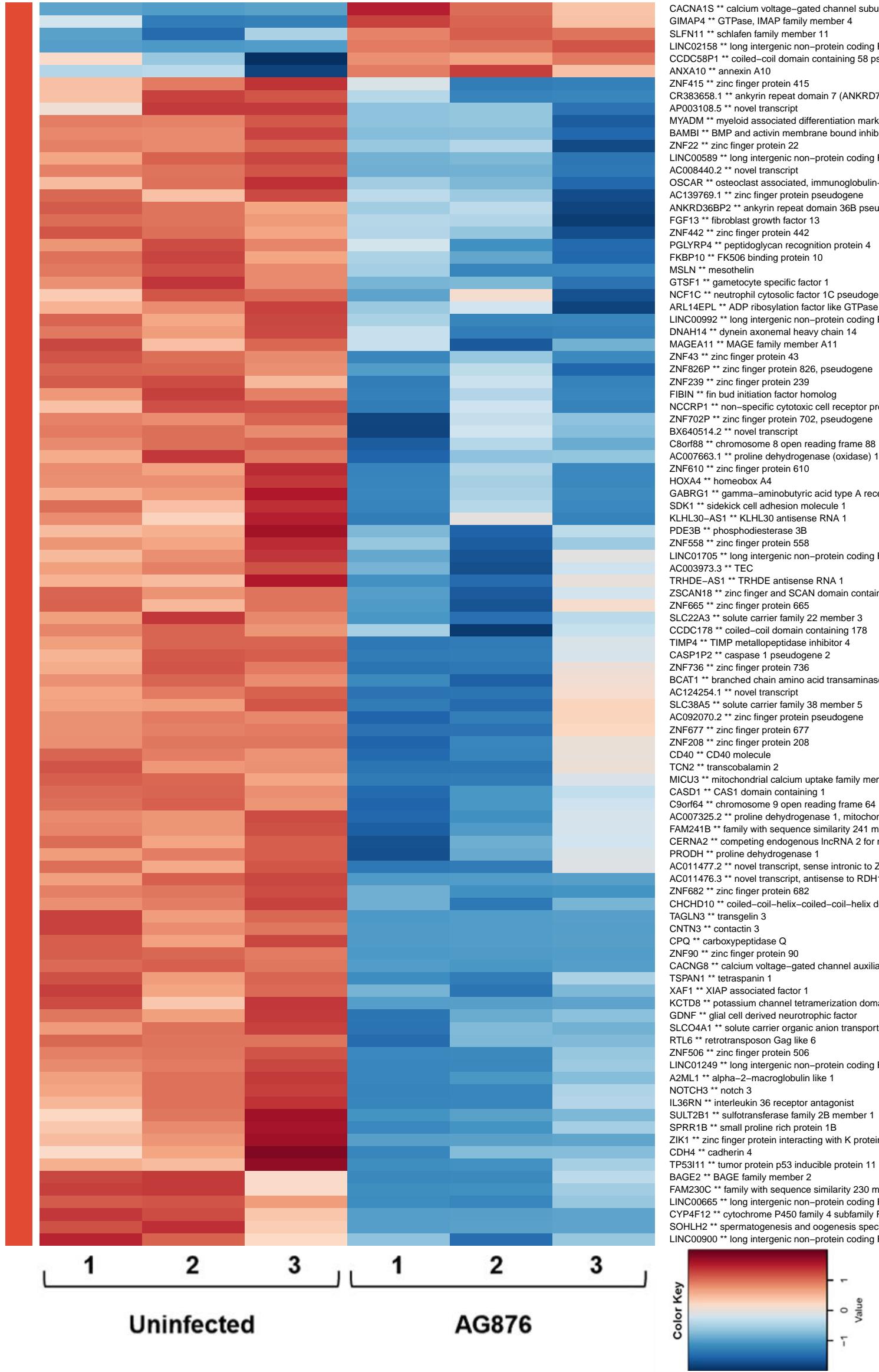

Supplement: S2 Fig — The top 100 differentially expressed cellular genes in the RNA-seq analysis are shown. Names for each cell line, as well as the EBV type and strain are shown. EBV-infected cells were all derived from the “NOKs-1” line and the uninfected NOKs were a mixture of two NOKs-1 samples and one NOKs-2 sample as indicated in S1 Table. Red indicates a gene is upregulated in corresponding cells and blue indicates it is down-regulated. (PDF) [file ppat.1010868.s002.pdf]

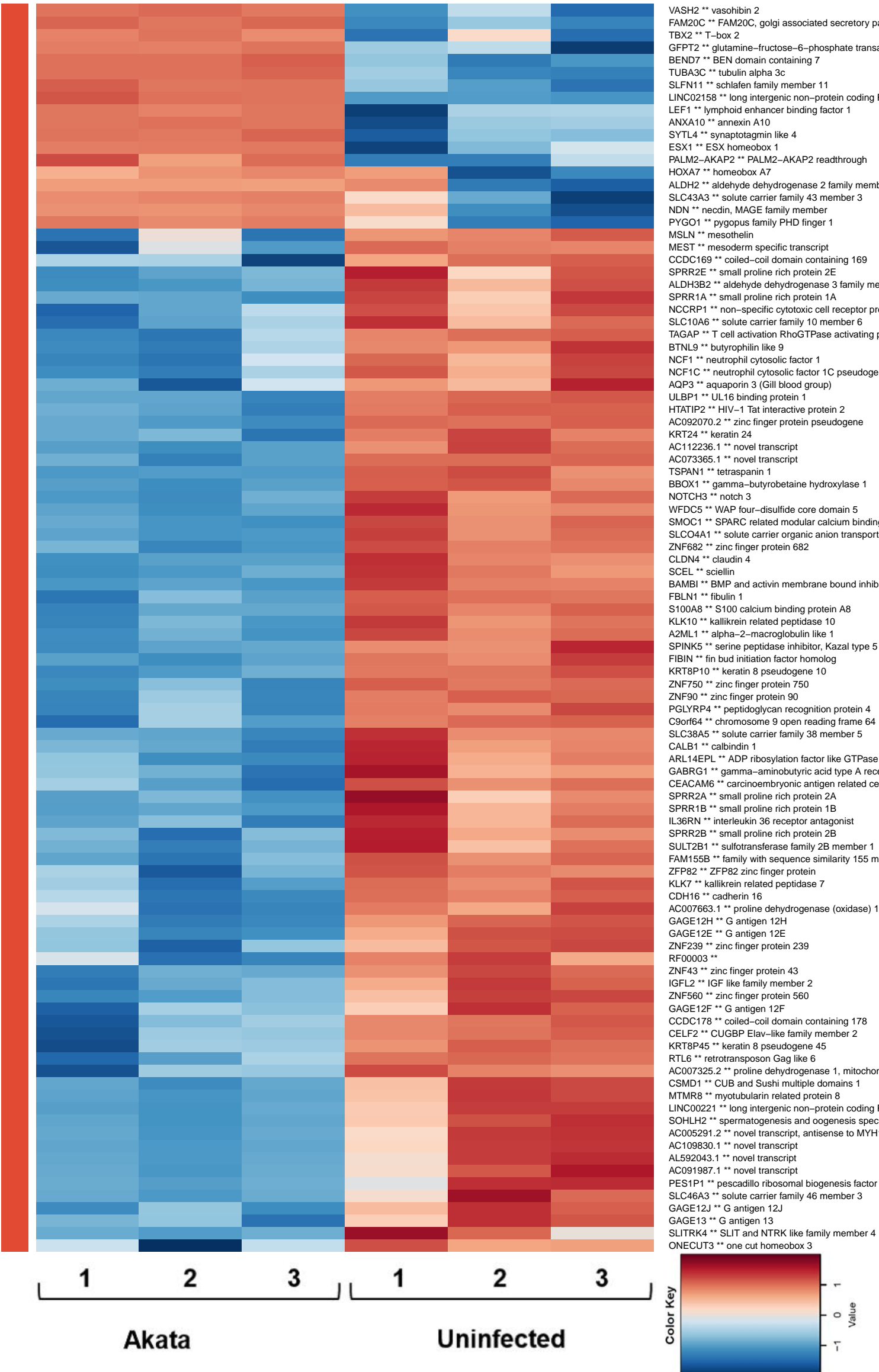

Supplement: S3 Fig — The top 100 differentially expressed cellular genes in the RNA-seq analysis are shown. Names for each cell line, as well as the EBV type and strain are shown. EBV-infected cells were all derived from the “NOKs-1” line and the uninfected NOKs were a mixture of two NOKs-1 samples and one NOKs-2 sample as indicated in S1 Table. Red indicates a gene is upregulated in corresponding cells and blue indicates it is down-regulated. (PDF) [file ppat.1010868.s003.pdf]

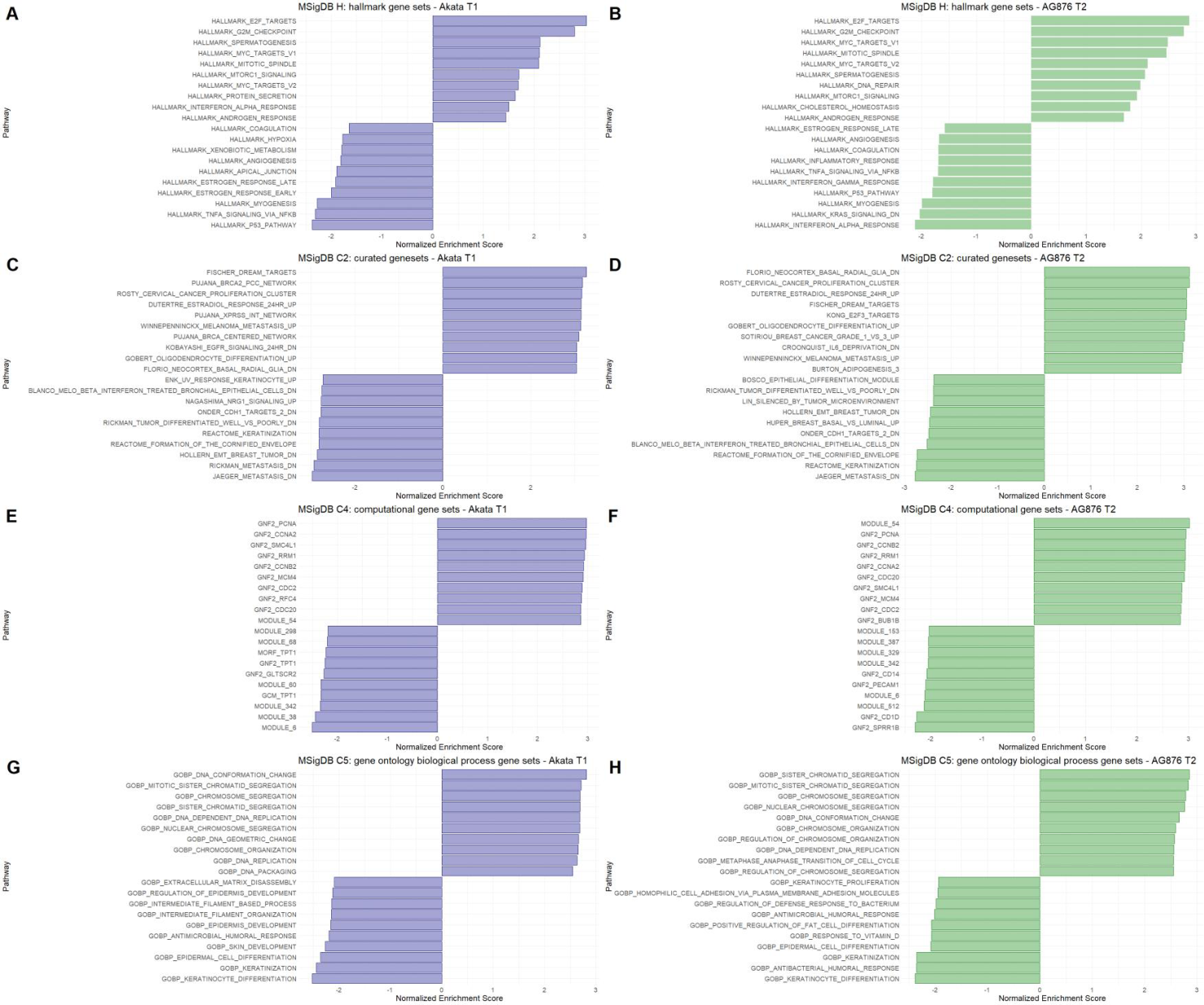

Supplement: S4 Fig — Results from GSEA for RNA-seq data of (A, C, E, G) Akata T1 or (B, D, F, H) AG876 T2 virus-infected NOKs versus uninfected NOKs as described in the Materials and Methods sections. Gene sets from the following MSigDB collections were analyzed: (A-B) hallmark, (C-D) curated, (E-F) computational, (G-H) and ontology sub-collection gene ontology biological process. Each enrichment plots display the top 10 up-regulated and top 10 down-regulated pathways with Benjamini-Hocheberg (BH)-adjusted p values of < 0.05 and were sorted by Normalized Enrichment Scores (NES). Pathways upregulated in the EBV-infected NOKs relative to uninfected NOKs are associated with NES values greater than 0, and down-regulated pathways are associated with NES values less than 0. (TIF) [file ppat.1010868.s004.tif]

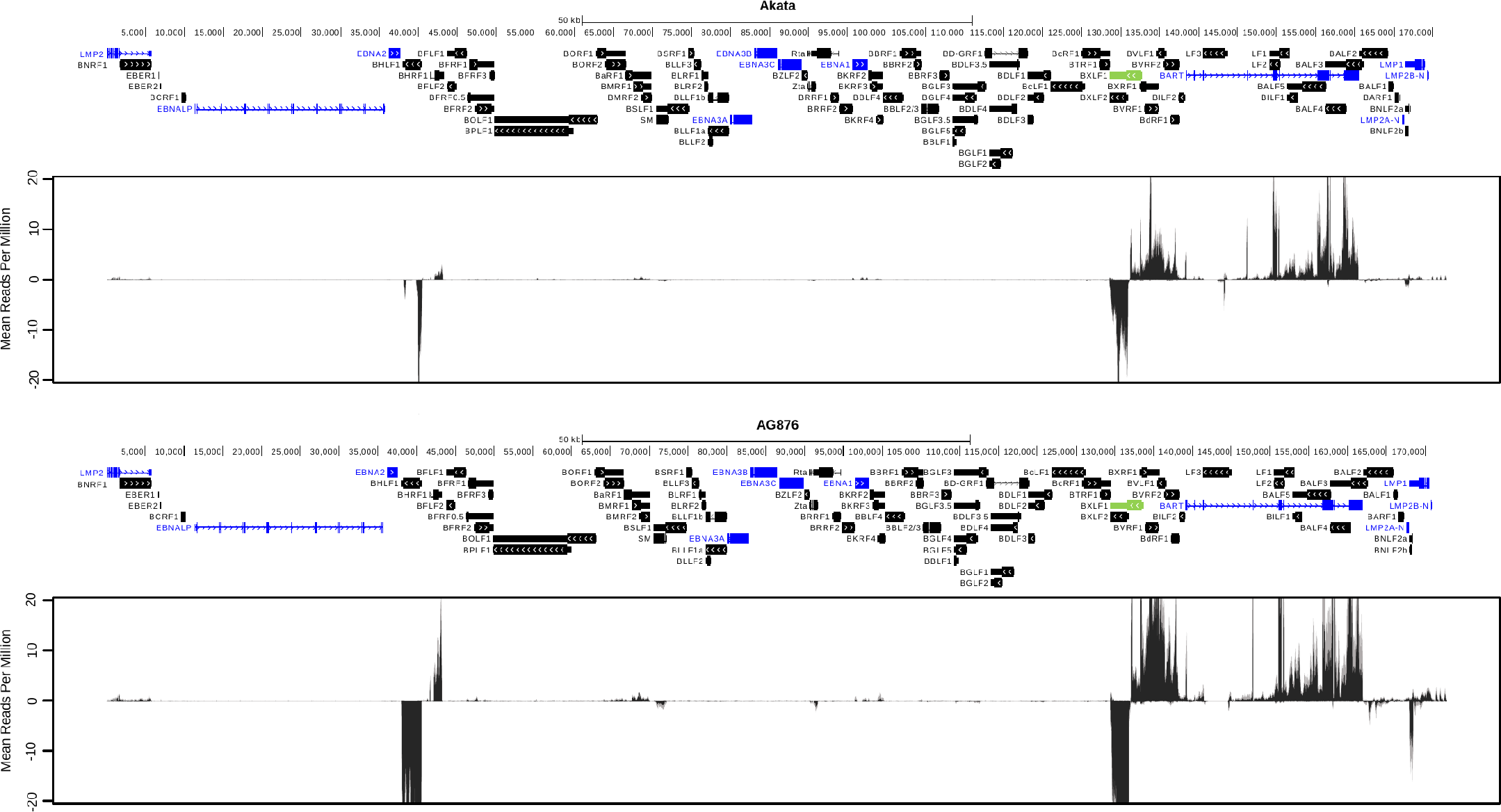

Supplement: S5 Fig — RNA-seq reads from NOKs cells infected with T1 or T2 viruses were aligned to the T1 or T2 EBV genomes, respectively. For each strain, wiggle tracks of normalized read depth were normalized and plotted (black) with standard errors (gray). Annotation tracks for type 1 and type 2 viruses showing latent (blue) genes and lytic (black) genes were generated using UCSC genome browsers and displayed above. The BXLF1 gene (shown in green) was disrupted by insertion of a G418R/GFP cassette and transcription in this region arises as a result of the promoters within this cassette. (TIF) [file ppat.1010868.s005.tif]

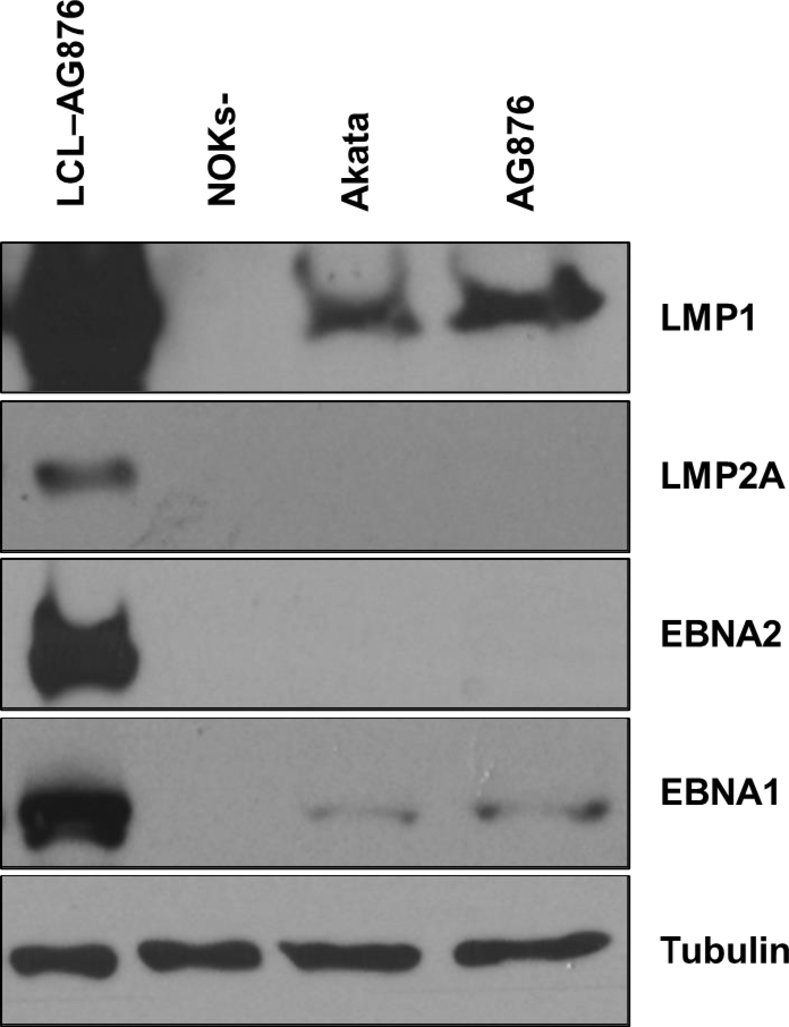

Supplement: S6 Fig — The expression levels of LMP1, LMP2A, EBNA2, and EBNA1 in uninfected, Akata virus infected and AG876 virus infected NOKs cells (each in the context of the “NOKs 2” line) was examined by immunoblot analysis. LCL-AG876 was also included in each blot and served as a positive control for all the EBV latent proteins probed here. Actin was also measured as a loading control. (TIF) [file ppat.1010868.s006.tif]

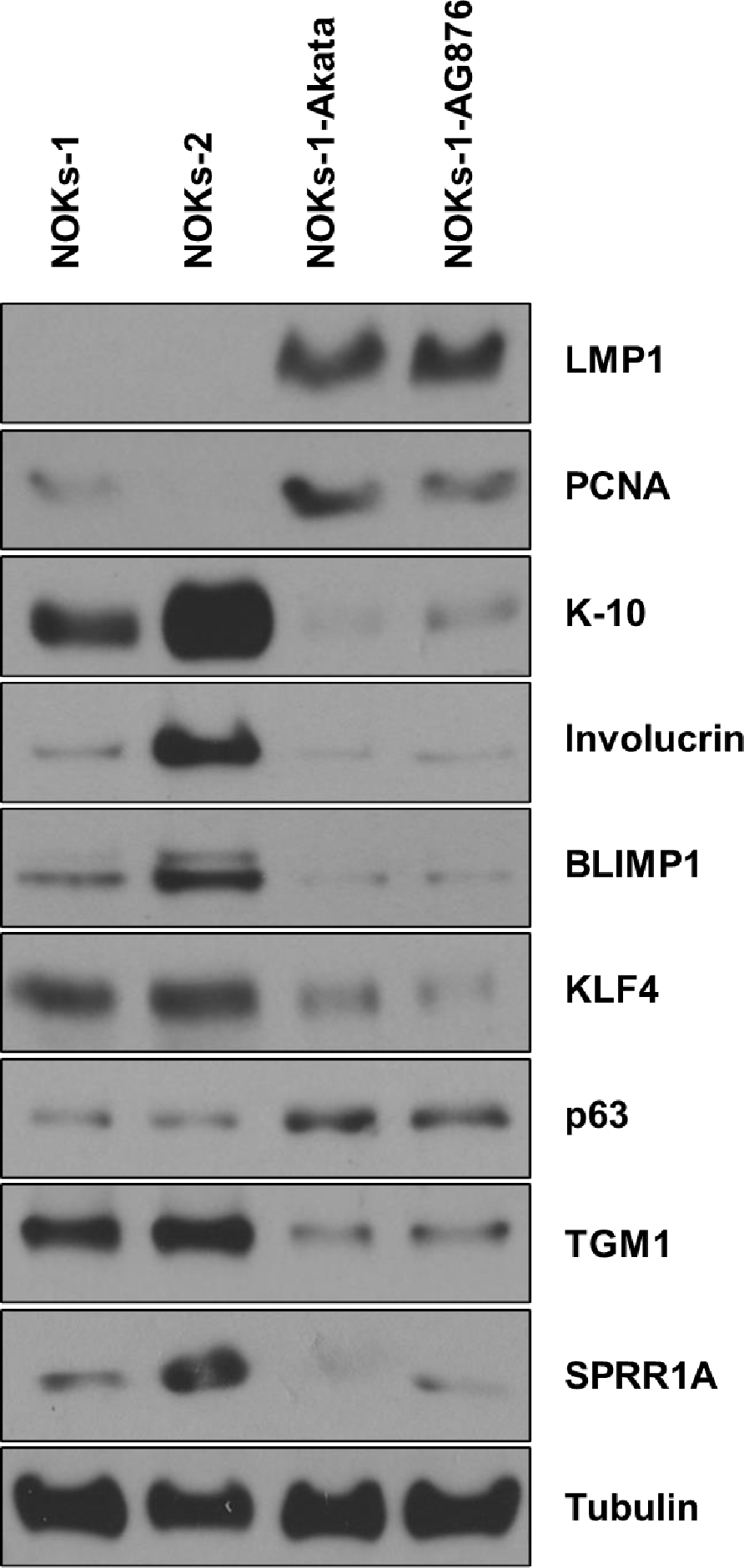

Supplement: S7 Fig — Uninfected NOKs (either the “NOKs-1” line or the “NOKs-2” line as indicated), or Akata EBV-infected, or AG876 EBV-infected NOKs (both in the context of the “NOKs-1” line) were seeded (125K cells per well) in 6 well plates and grown in KSFM medium without supplements for 24 hours. Immunoblot analysis was then performed to assess expression levels of LMP1, PCNA, Keratin-10 (K-10), Involucrin, BLIMP1, KLF4, delta p63, TGM1, SPRR1A or tubulin (loading control) as indicated. (TIF) [file ppat.1010868.s007.tif]

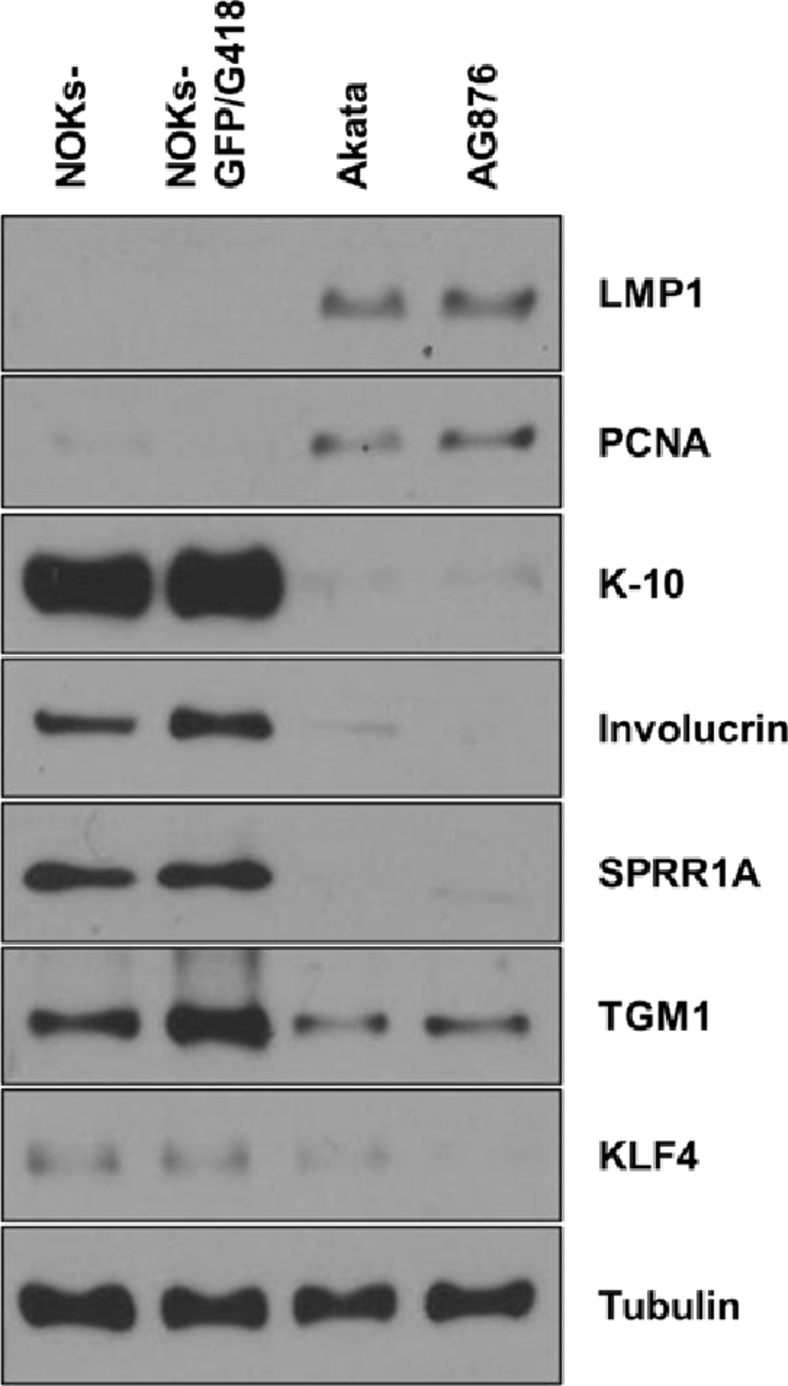

Supplement: S8 Fig — Uninfected (“NOKs-2”), NOKs-2 cells stably transfected a vector expressing the GFP and G418 resistance genes (GFP/G418R), on NOKs-2 cells infected with Akata EBV or AG876 EBV were seeded (125K cells per well) in 6 well plates and grown in KSFM medium without supplements for 24 hours. Immunoblot analysis was then performed to assess expression levels of LMP1, PCNA, Keratin-10 (K-10), Involucrin, SPRR1A, TGM1, KLF4 or tubulin (loading control) as indicated. (TIF) [file ppat.1010868.s008.tif]

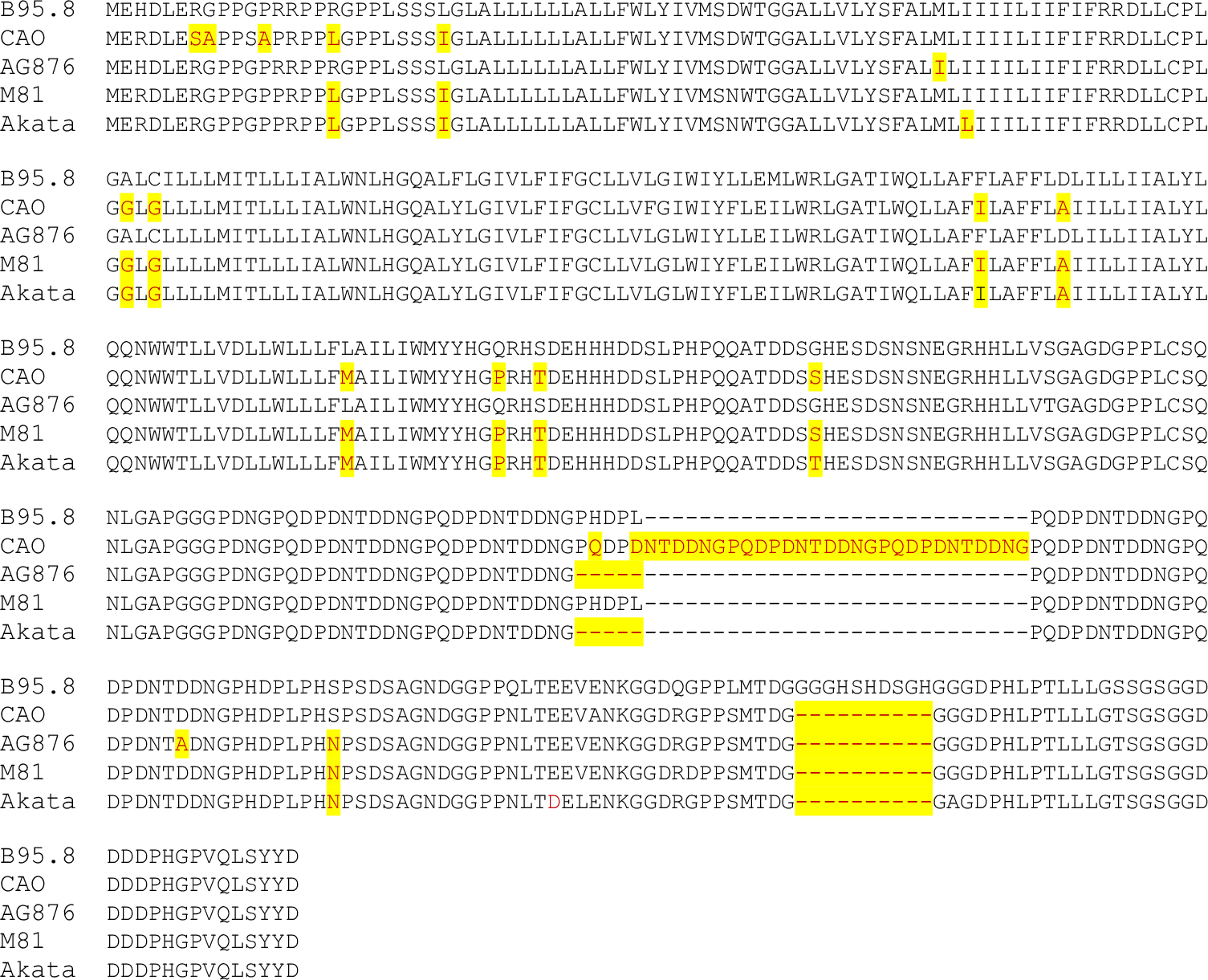

Supplement: S9 Fig — The LMP1 sequences of B95.8, CAO, AG876, M81, and Akata strains of EBV are compared. Amino acids that are different between strains are highlighted in yellow. (TIF) [file ppat.1010868.s009.tif]
